# Investigating the effects of single-dose intranasal testosterone on economic preferences in a large randomized trial of men

**DOI:** 10.1073/pnas.2508519122

**Published:** 2025-09-23

**Authors:** Anna Dreber, Magnus Johannesson, Gideon Nave, Coren L. Apicella, Shawn N. Geniole, Taisuke Imai, Erik L. Knight, Dylan Manfredi, Pranjal H. Mehta, Valentina Proietti, Steven J. Stanton, Alina Zeltikova, Francesca R. Luberti, Triana Ortiz, Justin M. Carré

**Affiliations:** ^a^Department of Economics, Stockholm School of Economics, Stockholm 113 83, Sweden; ^b^Department of Economics, University of Innsbruck, Innsbruck 6020, Austria; ^c^Marketing Department, Wharton School, University of Pennsylvania, Philadelphia, PA 19104; ^d^Department of Psychology, University of Pennsylvania, Philadelphia, PA 19104; ^e^Department of Psychology, University of the Fraser Valley, Abbotsford, BC V2S 7M8, Canada; ^f^Institute of Social and Economic Research, The University of Osaka, Osaka 567-0047, Japan; ^g^Department of Psychology and Neuroscience, University of Colorado, Boulder, CO 80309; ^h^Department of Experimental Psychology, University College London, London WC1E 6BT, United Kingdom; ^i^Department of Management and Marketing, School of Business Administration, Oakland University, Rochester, MI 48309; ^j^Department of Economics, University College London, London WC1H 0AN, United Kingdom; ^k^Department of Psychology, Nipissing University, North Bay, ON P1B 8L7, Canada

**Keywords:** testosterone, economic preferences, replications

## Abstract

There is conflicting evidence on whether testosterone affects economic preferences such as risk taking, fairness, and altruism, with most evidence coming from correlational studies or small testosterone administration studies. To credibly test this hypothesis, we conducted a large-scale, preregistered, double-blind randomized controlled trial with 1,000 male participants—10 to 20 times larger than typical prior studies. Participants were randomly allocated to receive a single dose of either placebo or intranasal testosterone, and carried out a series of economic tasks capturing social preferences, competitiveness, and risk preferences. We find no evidence of a treatment effect for any of our nine primary outcome measures, and no strong evidence of an association between basal salivary testosterone and economic preferences within men. These results fail to conceptually replicate previous high-impact publications reporting positive findings in smaller samples, calling into question the idea that short-term testosterone fluctuations are important drivers of men’s economic preferences. Our results do not rule out the possibility that different effects might emerge under alternative dosages, administration protocols, or task timings, or that behavioral effects differ between men and women. The potential for developmental or long-term effects of testosterone also remains an open question for future research, though such effects are ethically challenging to investigate experimentally in humans.

## Significance

Testosterone has been proposed to influence economic preferences such as risk taking, fairness, altruism, and competitiveness, with support from correlational studies and small-sample randomized trials. To rigorously test this hypothesis, we conducted a large-scale, preregistered, double-blind randomized controlled trial with 1,000 male participants—an order of magnitude larger than most prior experiments. Participants were randomly assigned to receive either a placebo or a single dose of intranasal testosterone and completed tasks measuring social preferences, competitiveness, and risk taking. We found no evidence that testosterone affected behavior in any of these domains, challenging the view that short-term exogenous testosterone fluctuations meaningfully shape men’s economic preferences. Developmental or long-term effects remain open questions.

Testosterone is typically referred to as the male sex hormone, although it is also present in women at much lower levels (1). Testosterone plays a vital role in the male reproductive system and is also related to masculinization effects such as increased muscle mass and strength, a deeper voice, and body hair growth (2–5). There is extensive literature attempting to link testosterone to behaviors such as aggression, dominance, and mate-seeking (6–11). In the last 15 y or so, we have also seen an increasing literature on the potential effects of testosterone on economic preferences and decision-making (e.g., refs. 12–20). Economic preferences vary substantially between individuals (21), and testosterone may potentially play a role in explaining some of this variability. It is intuitive that there could be a link from effects on masculinity, aggression, and dominance to economic behavior such as risk taking and the punishment of unfair behavior. Anger, for instance, has been linked to the rejection of unfair offers in the ultimatum game (22), and a recent meta-analysis found that anger increases economic risk-taking (23).

Economic games and incentivized measures are often used to study economic preferences (21). When it comes to social preferences, some of the most commonly deployed games are the dictator game, the ultimatum game, and the trust game. To study risk preferences, measures often involve participants choosing between a safe option and a lottery or between lotteries that differ in probabilities and potential outcomes. Competitiveness, or willingness to compete, is typically measured in the economics literature by having participants choose between performing a task under a piece-rate scheme or a tournament scheme. Behaviors in these games and tasks have, to various degrees, been linked to other real-world outcomes such as educational and career choices (24).

Economic preferences have also been linked to testosterone, through both correlational studies examining endogenous levels and studies of experiments that exogenously manipulate testosterone levels. See *SI Appendix*, Table S1 for a summary of key results in the previous literature for the economic preferences included in our study. Burnham (25) conducted an early correlational study reporting that men who rejected unfair offers had relatively higher baseline testosterone concentrations relative to men who accepted unfair offers in the ultimatum game. Other early correlational studies focused on risk-taking with substantial variation in results across studies. For example, Apicella et al. (26) found a positive correlation between testosterone and risk-taking in men; Sapienza et al. (27) found a positive correlation for women but not for men; Stanton et al. (28) found a U-shaped relationship, whereby men and women with intermediate levels of testosterone were risk and ambiguity averse and those with low/high levels of testosterone were risk and ambiguity neutral; and Schipper (29) found a positive correlation between testosterone and risk taking for men but not for women. The correlational results on dictator game giving (often studied as a proxy of altruism) are also conflicting. For example, Novakova et al. (30) found a negative correlation between testosterone and dictator game giving among women and a positive correlation among men. Eisenegger et al. (31) reported a positive correlation between testosterone and the willingness to compete.

The evidence from more controlled studies, exogenously manipulating testosterone using double-blind within or between-subjects designs, is also mixed. Zak et al. (12) reported that exogenous testosterone decreased offers and generosity in the ultimatum game in a sample of men. Eisenegger et al. (13), on the other hand, reported an increase in offers after testosterone administration in a sample of women. Geniole et al. (14) found no main effect of testosterone on ultimatum game offers in a sample of men, but reported an interaction with responder appearance. Dreher et al. (15) reported that testosterone did not affect ultímatum game rejections, but increased negative and positive reciprocity in men, whereas Boksem et al. (16) reported that testosterone decreased trust and increased trustworthiness in the trust game in women. In experimental asset market experiments, Cueva et al. (17) reported that testosterone increased investment for high variance stocks in men, and Nadler et al. (32) reported that testosterone led to larger and longer-lasting bubbles in men. In experiments with women, Van Honk et al. (18) and Buskens et al. (19) reported no main effects of testosterone but interaction effects between testosterone and 2D:4D (a putative proxy of prenatal testosterone exposure, though see ref. 33) in the public goods game and the trust game, respectively. Several studies have also reported null results for some of these measures and games (20, 34–37). Importantly, most of these studies have small samples. The number of participants in the studies reporting any statistically significant effects has ranged from *N* = 24 (18) to *N* = 118 (14). It is well known that small, underpowered studies increase the risk that findings reported as statistically significant are false positives (38–40). Many of these studies also lacked a proper preregistered analysis plan (41), increasing the risk of “researcher degrees of freedom” and selective reporting of statistically significant results that may systematically bias findings (42–45).

Given the conflicting evidence, it is crucial to provide further evidence from preregistered, high-powered, double-blind randomized controlled studies. We provide such evidence in an experiment of *N* = 1,000 male participants who were randomly allocated to receive a single dose of 11 mg intranasal testosterone gel or placebo in a double-blind design and, after a 30-min soak/absorption period, a time corresponding to peak testosterone concentrations (see ref. 46), completed a series of economic experiments and other tasks. This testosterone administration paradigm yields an increase in serum testosterone concentrations of 60 to 80% (46), and has been used in several previous studies (14, 47–50). We measure a series of economic preference tasks and test nine primary hypotheses based on preregistered hypothesis tests. We have 80% statistical power to detect a Cohen’s *d* effect size of 0.23 at the *P* < 0.005 threshold for statistical significance used in the study. Importantly, our research design only allows for tests of the causal effect of short-term fluctuations of testosterone on economic preferences and does not test for long-term or developmental effects. Our tests are also limited to men and may be sensitive to the exact dosage, testosterone administration used, and the timing of the measurement of economic preferences. Caution is thus required in generalizing our findings beyond the exact design and population used in our randomized study.

## Results

We use two-tailed tests in all tests below. As preregistered, we interpret results with *P* < 0.005 as “statistically significant evidence” and results with 0.005 ≤ *P* < 0.05 as “suggestive evidence,” as recommended by Benjamin et al. (51). We posted separate preanalysis plans (PAPs) for the following tasks; the ultimatum game (52) (https://osf.io/gtyk5/), the trust game (53) (https://osf.io/fc9ka/), the dictator game (54) (https://osf.io/6cqbh/), the charity game (53, 54) (https://osf.io/ua7nj/), a competition task (55) (https://osf.io/z7ary/), and a risk task (56–58) (https://osf.io/3emdr/) after data collection had started but before data were accessed or analyzed (data collection started in February 2018 and went on until December 2023, while the preanalysis plans were posted in March–June 2018). The preregistered analyses and tests were divided into hypotheses (which we will refer to as the primary hypotheses), robustness tests, and exploratory analyses. We followed the PAPs exactly except for some deviations listed in *Methods*; these deviations are due to ambiguity about the exact implementation of some of the tests and some differences, compared to the preregistration, in how some of the independent variables were collected and coded in the raw data. We also added one exploratory analysis of an additional outcome measure for risk attitudes, which was not part of the preregistration, and four sets of robustness tests and exploratory analyses described below that were not part of the preregistration.

All primary hypotheses were evaluated with a two-sample *t* test (for continuous variables) or a two-sample z-test for a difference in proportions, except one hypothesis involving an interaction in a linear mixed-effect model. For more details, see *Methods* and *SI Appendix*, sections 1–6. Due to logistical problems with the server used to implement the risk task, there were missing data on 46 participants on the risk task, affecting the two primary outcome measures, Risk Aversion and Loss Aversion. There are also some missing observations on other variables, as reflected by the number of observations reported in the results tables. In *SI Appendix*, Tables S2 and S3, we report descriptive statistics for the participants in each group at baseline and for the outcome variables and independent variables measured after the intervention.

### Manipulation Check.

One thousand male participants completed the study, with *N* = 500 in the placebo group and *N* = 500 in the testosterone group. As a manipulation check, testosterone was measured in saliva samples in both groups 30 min after the testosterone administration (before the start of the tasks used as outcome measures in the study). Postadministration salivary testosterone levels are statistically significantly higher in the testosterone group than in the placebo group (independent sample *t* test of the log of testosterone: *t*(993) = 37.07, *P* < 0.0001; *n* = 995); this manipulation check test was not preregistered. The magnitude of the testosterone increase is difficult to assess in saliva samples and the saliva samples should only be interpreted as showing that testosterone was higher in the testosterone group than in the placebo group. Specifically, postnasal drip may lead to some exogenous testosterone gel making its way directly from the nasal cavity into one’s mouth, and thus, making it difficult to quantify with precision the true magnitude of the rise in testosterone that occurs after administration. Nevertheless, previous work has demonstrated that the testosterone administration paradigm used in this experiment reliably produces a 60 to 80% rise in serum testosterone concentrations (46, 47).

### Effect of Testosterone On Economic Preferences.

**Fig. 1.**
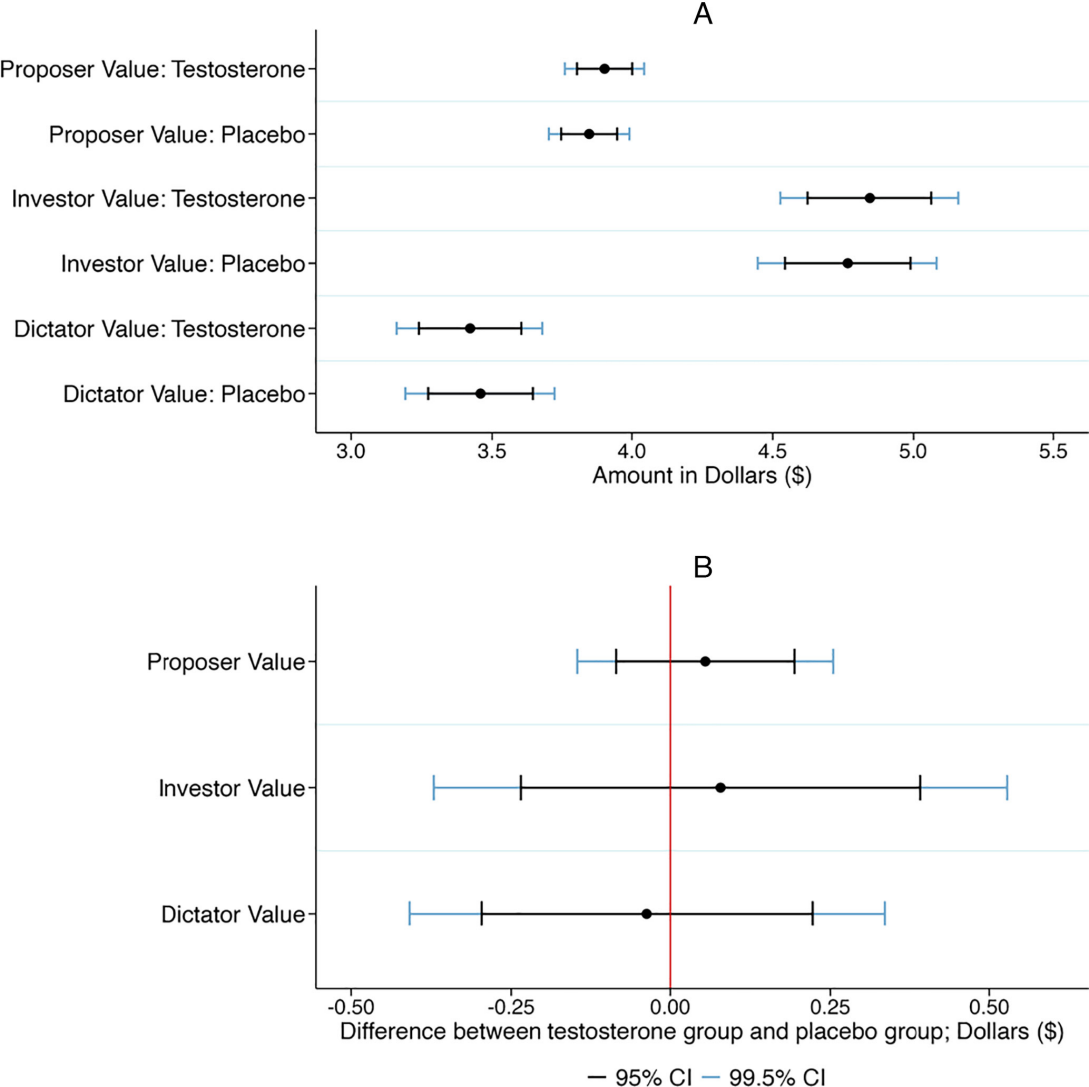
Treatment effects of testosterone on Proposer Value in the ultimatum game, Investor Value in the trust game, and Dictator Value in the dictator game. (*A*) Plotted are the 95% and 99.5% CI of the Proposer Value, the Investor Value, and the Dictator Value in the testosterone group and the placebo group. (*B*) Plotted are the 95% and 99.5% CI of the treatment effects on Proposer Value (hypothesized positive effect of testosterone), Investor Value (hypothesized negative effect of testosterone), and Dictator Value (hypothesized negative effect of testosterone). Positive treatment effects imply a positive effect of testosterone on the outcome variable. We cannot reject the null hypothesis of no treatment effect for any of these three variables; see Table 1 for detailed test results.

**Fig. 2.**
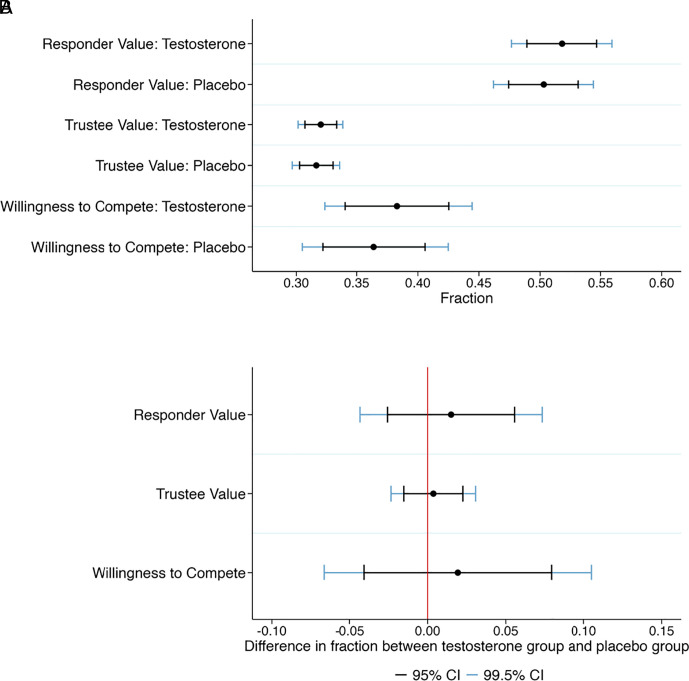
Treatment effects of testosterone on Responder Value in the ultimatum game, Trustee Value in the trust game, and Willingness to Compete. (*A*) Plotted are the 95% and 99.5% CI of the Responder Value (fraction of rejection of unfair offers), the Trustee Value (fraction of tripled investment backtransferred), and the Willingness to Compete (fraction choosing competitive payment scheme) in the testosterone group and the placebo group. (*B*) Plotted are the 95% and 99.5% CI of the treatment effects on Responder Value (hypothesized positive effect of testosterone), Trustee Value (hypothesized positive effect of testosterone), and Willingness to Compete (hypothesized positive effect of testosterone). Positive treatment effects imply a positive effect of testosterone on the outcome variable. We cannot reject the null hypothesis of no treatment effect for any of these three variables; see Table 1 for detailed test results.

**Fig. 3.**
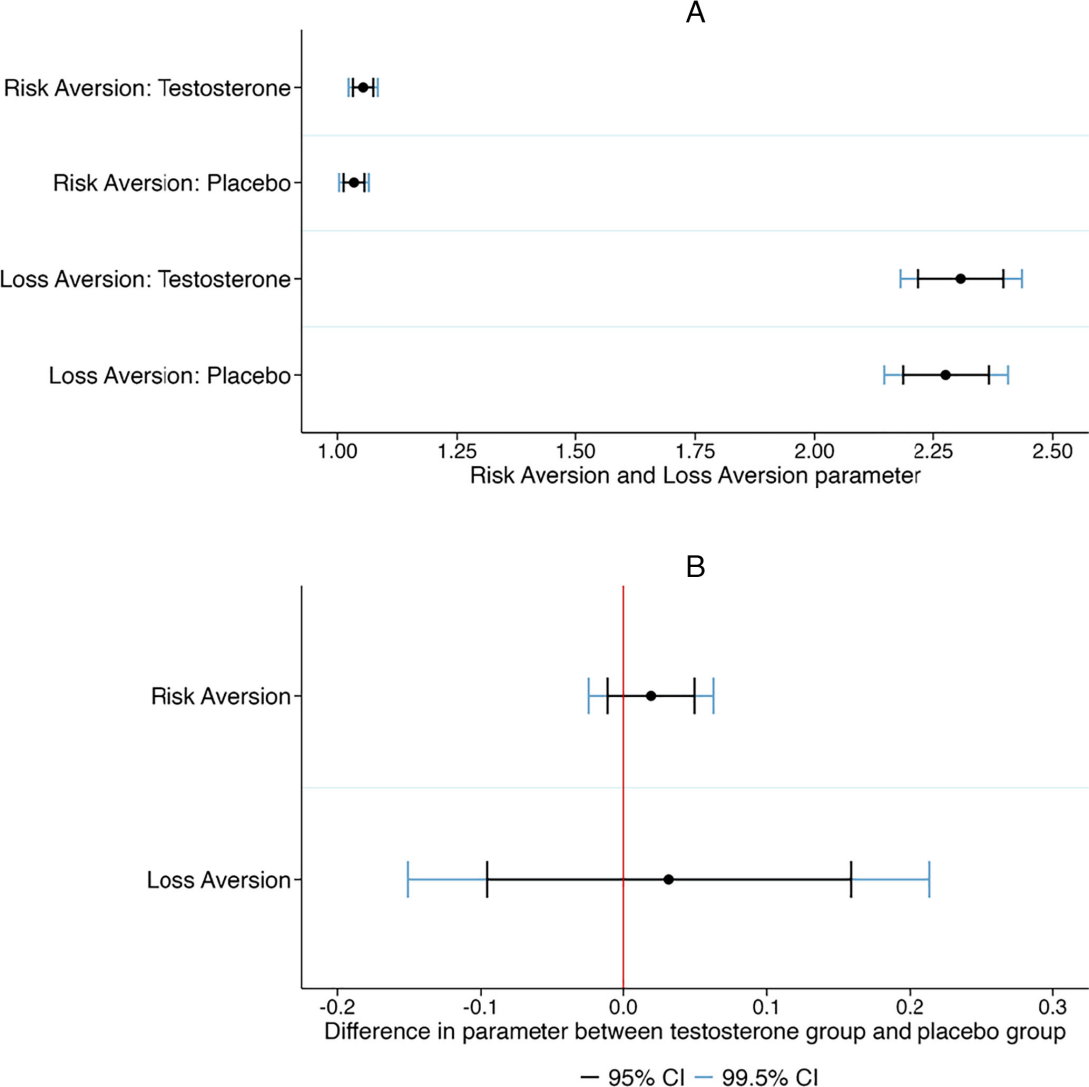
Treatment effects of testosterone on Risk Aversion and Loss Aversion. (*A*) Plotted are the 95% and 99.5% CI of the estimated Risk Aversion parameter and Loss Aversion parameter in the testosterone group and the placebo group. (*B*) Plotted are the 95% and 99.5% CI of the treatment effects on Risk Aversion (hypothesized negative effect of testosterone) and Loss Aversion (hypothesized negative effect of testosterone). Positive treatment effects imply a positive effect of testosterone on the outcome variable (although note that a higher Risk Aversion parameter implies less risk aversion). We cannot reject the null hypothesis of no treatment effect for any of these three variables; see Table 1 for detailed test results.

We describe our nine primary hypotheses in Table 1. The first eight primary hypotheses tested for the main effect of testosterone on Risk Aversion, Loss Aversion, Proposer Value, Responder Value, Investor Value, Trustee Value, Dictator Value, and Willingness to Compete. See Table 1 for the preregistered hypothesized directions of these eight hypotheses, with the directions of the predictions based on reported positive findings in previous studies. In Figs. 1–3, we show the 95% and 99.5% CI of these eight outcome variables in the testosterone group and the placebo group (panel *A*) and the CI of the treatment effects (panel *B*). The behavior in the economic preference tasks, such as the ultimatum game and the trust game, are in line with what is typically observed for these games (21). We cannot reject the null hypothesis of no difference between the testosterone and the placebo groups at either the 0.5% (statistically significant evidence) or the 5% (suggestive evidence) level in any of the eight outcome measures. The exact hypothesis test results are reported in Table 2.

**Table 1.** Primary hypotheses tested in the study

Hypothesis	Outcome variable	Definition of the outcome variable	Hypothesized sign
Testosterone leads to more generous offers in the ultimatum game.	Proposer Value	The proposal to the responder in the ultimatum game ($0 to $10).	Positive
Testosterone leads to more rejections of unfair offers in the ultimatum game.	Responder Value	The fraction of unfair offers rejected by the responder in the ultimatum game (0 to 1).	Positive
Testosterone leads to lower investments in the trust game.	Investor Value	The amount invested in the trust game ($0 to $10).	Negative
Testosterone leads to larger backtransfers (reciprocity) in the trust game.	Trustee Value	The fraction of the tripled amount invested that is backtransferred in the trust game (0 to 1).	Positive
Testosterone leads to less generosity in the dictator game.	Dictator Value	The amount given to the recipient in the dictator game ($0 to $10).	Negative
Testosterone leads to more willingness to compete.	Willingness to Compete	The choice of a competitive payment scheme rather than a piece rate (1/0 variable).	Positive
Testosterone leads to less risk aversion.^*^	Risk Aversion	The estimated risk aversion parameter in the risk task (a higher parameter implies less risk aversion).	Positive
Testosterone leads to less loss aversion.	Loss Aversion	The estimated loss aversion parameter in the risk task.	Negative
Testosterone leads to less sensitivity to the price of donating to charity.^†^	Percentage Donated	The percentage donated to charity out of the endowment (0 to 100%).	Positive

^*^Note that a higher risk aversion parameter implies less risk aversion and the hypothesized positive sign thus implies that testosterone is hypothesized to decrease risk aversion.

^†^This hypothesis is tested by a “Price of Donating*Treatment interaction” variable in a linear mixed-effect model with Percentage Donated as the dependent variable.

**Table 2.** Test results of the nine primary hypotheses

Outcome variable	Hypothesized sign	n	Treatment effect (SE)	95% CI (99.5% CI)	t/z-value (df)	*P*-value
Proposer Value	Positive	999	0.055 (0.071)	−0.085; 0.194 (−0.145; 0.255)	0.770 (997)	0.441
Responder Value	Positive	999	0.015 (0.021)	−0.026; 0.056 (−0.043; 0.073)	0.724 (997)	0.469
Investor Value	Negative	999	0.078 (0.160)	−0.235; 0.392 (−0.371; 0.528)	0.491 (997)	0.623
Trustee Value	Positive	999	0.0036 (0.0096)	−0.015; 0.022 (−0.023; 0.031)	0.380 (997)	0.704
Dictator Value	Negative	999	−0.037 (0.132)	−0.296; 0.223 (−0.409; 0.335)	−0.279 (997)	0.780
Willingness to Compete	Positive	999	0.019 (0.031)	−0.041; 0.079 (−0.067; 0.105)	0.630	0.528
Risk Aversion^*^	Positive	954	0.019 (0.015)	−0.011; 0.050 (−0.024; 0.063)	1.248 (952)	0.212
Loss Aversion	Negative	954	0.032 (0.065)	−0.096; 0.159 (−0.151; 0.214)	0.486 (952)	0.627
Percentage Donated^†^	Positive	11,988	−1.908 (2.560)	−6.924; 3.108 (−9.100; 5.284)	−0.745 (997)	0.456

^*^Note that a higher risk aversion parameter implies less risk aversion and the hypothesized positive sign thus implies that testosterone is hypothesized to decrease risk aversion.

^†^This hypothesis is tested by a “Price of Donating*Treatment interaction” variable in a linear mixed-effect model with Percentage Donated as the dependent variable. Every participant is included with 12 observations in the regression explaining the high n.

In a final primary hypothesis test, we tested whether testosterone leads to less sensitivity to the cost of donating to charity. This hypothesis is motivated by the idea that testosterone increases status-seeking behavior (7) and that the status benefits of altruistic behavior increase if it is more costly, as found by Hardy and Van Vugt (59). To test this, we implemented a charity game where participants decided how much to donate to a charity out of an endowment while varying the multiplier of the donation [as in Eckel and Grossman (60, 61)]. The inverse of this multiplier is defined as the Price of Donating, and we tested whether this variable interacts with testosterone treatment in its effect on the Percentage Donated to charity. We find no evidence of this hypothesis either; see Table 2.

### Robustness Tests.

In preregistered robustness tests, we test the above nine hypotheses after controlling for right-hand 2D:4D, pleasant-unpleasant mood, arousal-calm mood, time of day, and data collection site (see *SI Appendix*, section 9 for definitions of these control variables). We control for mood to rule out that an eventual treatment effect is due to an effect of testosterone on mood, and control for the other variables as they could potentially explain some of the variation in the outcome measures and thereby increase statistical power (but note that the primary hypotheses tests are unbiased without including control variables due to the randomization). For Willingness to Compete, we also control for Risk Aversion and math ability in this robustness test, to rule out that an eventual treatment effect goes via an effect on these variables. Note that the number of observations is decreased in these robustness tests as there are missing data on right-hand 2D:4D for 92 participants. In the Willingness to Compete robustness test, the sample is further reduced due to missing data on Risk Aversion. We do not find statistically significant or suggestive evidence of an effect of testosterone in any of these robustness tests; see *SI Appendix*, Table S4 for more detailed results.

For the Proposer Value and Responder Value in the ultimatum game, we furthermore carry out one additional robustness test, adding a variable for the treatment belief to the above control variables (a variable denoting whether participants believed they had received testosterone or placebo, based on a survey after the experimental tasks). This robustness test is motivated by the findings of Eisenegger et al. (13), who reported that testosterone increased Proposer Value only after controlling for the treatment belief. We cannot confirm their findings and find no evidence of an effect of testosterone on Proposer Value after controlling for the treatment belief. In ref. 13, proposers who believed they had received testosterone made lower offers, and there is suggestive evidence of an association in the same direction in our data with somewhat lower offers for proposers who believed they had received testosterone (we did not preregister reporting this as a separate test but report it for completeness). However, the magnitude of the estimated effect of treatment belief on Proposer Value is only about 20% of the effect reported in ref. 13. Also, for the Responder Value tests, the above conclusions are unchanged after controlling for treatment belief (and we find no evidence of an association between treatment belief and Responder Value; we did not preregister reporting this as a separate test but report it for completeness). See *SI Appendix*, Table S5 for more detailed results.

### Preregistered Exploratory Analyses.

In an exploratory analysis, we test for a main treatment effect on the donations to charity in the charity game, and the impact of competition on performance in the Willingness to Compete task (the Performance Change Score). We find no evidence of a treatment effect in these tests either.

We also conducted several additional preregistered exploratory analyses. We test whether basal salivary testosterone levels are associated with the eight main effect primary outcome measures and the Performance Change Score (see Table 1 for the primary outcome measures). We find no evidence of an association for any of these outcome measures. For the same outcomes, we also test for an interaction between basal salivary testosterone and basal salivary cortisol, which have been argued to interact in affecting economic decision-making (62–64). We find no evidence of such an interaction in the hypothesized direction, but for the Performance Change Score, there is suggestive evidence of an association in the opposite direction of the hypothesis; this is likely a false positive finding due to the many tests conducted and as noted below this result is not robust to excluding outliers in basal salivary testosterone. For Willingness to Compete, Performance Change, Risk Aversion, and Loss Aversion, we also test for an association with basal salivary cortisol but find no evidence of such an association. For the same four outcome variables and Responder Value and Dictator Value we also test for an interaction between treatment and basal salivary cortisol, but again find no evidence of an association.

We also carry out exploratory analyses focusing on specific outcome measures. We test whether the association between treatment and Trustee Values is stronger for higher investment amounts, but find no evidence of this. For the analyses involving the Percentage Donated outcome variable, we test whether testosterone’s effects on price inflexibility are stronger in men with higher trait impulsivity (a three-way interaction) and whether testosterone’s effects on price inflexibility are stronger in men who believe they received placebo than in men who believe they received testosterone (a three-way interaction). We find no evidence of such three-way interactions. We also tested whether the association between treatment and Willingness to Compete was stronger for participants with higher trait dominance, but we found no evidence of such an interaction. Finally, we test whether the association between treatment and risk aversion is stronger for participants with greater self-construal but find no evidence of such an interaction. See *SI Appendix*, Tables S6–S11 for the detailed results of the exploratory analyses and the hypothesized directions of the tests and *SI Appendix*, sections 1–6 for more details about the tests.

### Non-Preregistered Exploratory analyses.

In connection with the risk assessment task, financial risk attitudes were also assessed on a 0 to 10 scale from completely unwilling (0) and completely willing (10) (65). In an exploratory analysis, we test for a treatment effect on this alternative indicator of risk-taking using an independent samples *t* test. We find no evidence of a treatment effect, see *SI Appendix*, section 7 and Table S12 for more details.

### Non-Preregistered Robustness Tests and Exploratory analyses.

Based on suggestions from reviewers, we also conduct four sets of additional tests that were not preregistered. In a first set of robustness tests, we add controls (dummy coded) for year and month of data collection in all our regression analyses. In the regressions not already including a control for time of day, we also add time of day as a control. These results, reported in *SI Appendix*, Tables S13–S20, do not affect the conclusions of our study with the exception of one analysis. There is now suggestive evidence of a positive correlation between basal salivary testosterone and Proposer Value, with the *P*-value changing from 0.060 to 0.047; but this result is not robust to excluding outliers (see below). In an additional set of robustness tests, we exclude outliers with basal salivary testosterone values (pg/mL) >150 (83 participants). Using salivary tests to measure basal testosterone is associated with lower reliability than using serum tests, which may explain the high SD in baseline salivary testosterone levels driven by some outliers. In this set of robustness tests, we re-estimate all the exploratory analyses testing for an effect of basal salivary testosterone (including the tests of interaction effects between basal salivary testosterone and other variables). The results are reported in *SI Appendix*, Tables S21–S25. The conclusions of these analyses are not affected by the inclusion or exclusion of these outliers, with the exception of one analysis. There is no longer suggestive evidence of an interaction between basal salivary testosterone and basal salivary cortisol in the opposite direction of the hypothesis for the Performance Change Score. As noted above there is also no longer suggestive evidence of an association between basal salivary testosterone and Proposer Value in line with the baseline results, but in contrast to the robustness test above controlling for time of day, year, and month of data collection. In the third set of tests, we test for nonlinearities in the exploratory analyses testing for an association between basal salivary testosterone and the outcome measures by adding the square of basal salivary testosterone to the regressions. We carry out these analyses both with and without the outliers in basal salivary testosterone and these results are reported in *SI Appendix*, Tables S26 and S27. There is no evidence of an effect of the squared term in any of these regressions, with the exception of one analysis. There is suggestive evidence of a nonlinear positive association between basal salivary testosterone and Willingness to Compete if the outliers are excluded (both the basal salivary testosterone coefficient and the coefficient of the squared variable are positive with *P*-values of 0.04). However, we would not give much weight of evidence for this association unless confirmed in additional preregistered studies. In the final set of tests, we test for heterogeneous treatment effects by adding an interaction between treatment and basal salivary testosterone to the exploratory analyses testing for an association between basal salivary testosterone and the outcome measures. We carry out these analyses both with and without the outliers in basal salivary testosterone and these results are reported in *SI Appendix*, Tables S28 and S29. We find no evidence of interactions in these analyses.

## Discussion

We fail to find evidence of a treatment effect of a single-dose of intranasal testosterone on any of our eight primary outcome measures or the hypothesized interaction effect for the ninth primary outcome measure. The 99.5% CI can be used to interpret which effect sizes in the hypothesized direction we find strong evidence against, see Figs. 1–3. For our eight main effects primary hypotheses, we find strong evidence against effect sizes between about 0.15 Cohen’s *d* units (Investor Value and Loss Aversion) and 0.26 Cohen’s *d* units (Risk Aversion), which are considered small effect sizes. We thus find strong evidence against the hypothesis that single-dose intranasal testosterone administration has important effects on economic preferences or behavior in men for all outcome measures in our study. This finding is corroborated by the lack of any strong evidence of a correlation between basal salivary testosterone and any of the primary outcome measures, and in these preregistered exploratory analyses we have 80% statistical power to detect a correlation of 0.12 at the 0.5% significance level.

Our study can be considered a highly powered conceptual replication of several previous results reported in high-impact journals, with a 10 to 20 times larger sample size than most previous randomized controlled studies. However, our study does not constitute a direct replication of any specific previous study as we did not base our design on one individual previous study (66). Our study fails to conceptually replicate the following previous findings about treatment effects of testosterone: that testosterone increases offers in the ultimatum game (13); that testosterone decreases offers in the ultimatum game (12); that testosterone decreases trust in the trust game (16); that testosterone increases trustworthiness (backtransfers) in the trust game (16), and that testosterone increases risk taking (17). We furthermore fail to conceptually replicate previous correlational results that testosterone is positively correlated with economic risk-taking (26, 29, 67), that testosterone is positively correlated with generosity in the dictator game (30), and that testosterone is positively correlated with the rejection of unfair offers in the ultimatum game (25). A previous study has also reported that testosterone is positively correlated with willingness to compete (27). We failed to conceptually replicate this association in our preregistered exploratory analyses, but in a non-preregistered exploratory analysis testing for nonlinear effects we found suggestive evidence of a nonlinear positive association if outliers in basal salivary testosterone are excluded. However, this association would need to be confirmed in additional preregistered tests to carry much weight. In addition, testosterone and cortisol have been argued to interact in affecting economic decision-making with some reported significant interactions (62–64), but we find no evidence of interactions between basal salivary testosterone and basal salivary cortisol or testosterone treatment and salivary cortisol in the hypothesized directions.

Our study has important limitations that limit the generalizability of our results. First, we only included men, whereas Eisenegger et al. (13) and Boksem et al. (16) included only women in their studies. We cannot rule out that the effects of testosterone on economic preferences differ between men and women, and future research is needed to evaluate the generalizability of our findings to women. Another limitation is that the dosage, the administration protocol, and the timing of hormone administration may be important for observing an effect on economic preferences. Previous work has demonstrated that the testosterone administration paradigm used here yields an increase in serum testosterone concentrations of 60 to 80% within 30 min (46), and that this impacts outcome measures in men such as aggression and dehumanization (14, 47, 49, 50). Previous work also indicates that a single testosterone dose impacts threat-related amygdala function in both men (68) and women (69) within 45 to 60 min of administration, suggesting that testosterone may exert relatively rapid, possibly nongenomic, effects on human brain function. Such findings are consistent with a growing body of work in animal models demonstrating rapid effects of testosterone on several behavioral outcomes [see Nyby (70) for a review]. Nonetheless, it remains possible that a longer time lag [e.g., 3.5 to 4 h postadministration, as per Tuiten et al. (71)] may be required to observe effects of testosterone on economic preferences, although the testosterone level will have returned to the baseline level after 3.5 to 4 h. However, Tuiten et al. included only eight women participants and provided limited evidence of a treatment effect (*P* = 0.04), with outcomes measured after testosterone levels had already returned to baseline. Thus, while we cannot rule out delayed effects of single-dose testosterone on behavior, there is no conclusive evidence of this.

Importantly, our single-dose paradigm only causally investigates short-term fluctuations in testosterone. Administering testosterone over a longer period might affect economic preferences via neural, physiological, or psychological mechanisms that differ from the short-term effects studied here. It is, however, challenging to experimentally study such long-term effects in healthy individuals for practical and ethical reasons. We are aware of one previous study investigating the effects of longer testosterone exposure in a randomized and double-blind design. Zethraeus et al. (17) used a 4-wk administration period in a group of menopausal women comparing both testosterone and estrogen to placebo (*N* = 200). In line with our results, they found null results for a host of economic preference tasks, although with substantially lower statistical power to detect effects than in our study.

Testing for an association between basal testosterone and economic preferences can also provide some information about the impact of more long-term fluctuations in testosterone on economic preferences. However, such research designs do not provide causal evidence. The lack of any significant associations between basal salivary testosterone and economic preferences in our preregistered exploratory analyses is consistent with our null results for our primary treatment effects tests. In addition to being correlational, a further limitation of these analyses in our study is that the immunoassay salivary tests used to estimate the baseline testosterone levels are less reliable than using serum tests, but this is especially the case when hormone levels are very low, such as when measuring salivary testosterone in females (72, 73). In males, sizable positive associations between salivary testosterone measured with immunoassays and liquid chromatography–tandem mass spectrometry (LC–MS/MS) have been observed (72, 73). The high SD in the baseline salivary testosterone values driven by some large outliers may be due to the lower reliability in immunoassay tests. Our exploratory analyses testing for an association between basal salivary testosterone and economic preferences should therefore be interpreted very cautiously. Using serum tests, which require blood drawing, was not feasible in our study given the large sample size, and as our focus was on testing for treatment effects based on our randomized design rather than test for correlational evidence. This is also reflected in our preregistered hierarchy of tests with the correlational tests based on the baseline salivary testosterone levels preregistered as exploratory analyses. Note also that our correlational results are consistent with the recent large sample (*N* = 1,002) correlational study by Massaccesi et al. (74) who found no significant associations between testosterone and behavior in the dictator game, trust game, or ultimatum game based on measuring testosterone with LC–MS/MS in hair samples.

Our findings also do not address the potential impact of testosterone exposure during critical developmental periods, such as in utero or during puberty, when hormonal influences may play a role in shaping brain organization and long-term cognitive tendencies. These developmental windows may involve organizational effects that differ fundamentally from the short-term activational effects studied in our trial. However, experimentally investigating such developmental influences in humans is not feasible due to ethical constraints. As a result, existing evidence primarily relies on indirect and controversial proxies, such as studies of clinical populations or the second-to-fourth digit ratio (2D:4D), whose reliability and validity have been seriously questioned (33). Moreover, recent work that directly measured testosterone levels at birth using cord blood, alongside 2D:4D ratios, found no evidence that either metric predicted economic preferences later in life (75).

It is also possible that the treatment effect of testosterone on economic behavior is heterogeneous between individuals and interacts with other variables such as baseline hormones. Such heterogeneity can imply treatment effects in different directions in different subgroups, leading to an overall null effect on behavior. Based on previous literature, we tested for several interactions in our preregistered exploratory analyses, but found no strong evidence of such interactions. In non-preregistered robustness tests we furthermore tested for interactions between treatment and basal salivary testosterone, but found no evidence of such interactions either. Relatedly, there could be nonlinear effects between basal hormones and economic behavior, but we found no evidence suggesting this in non-preregistered robustness tests. Our data have been publicly posted at OSF, providing the possibility for other researchers to use our data for further exploratory analyses that can form the basis of future confirmatory tests in prospective preregistered studies.

The failure to conceptually replicate several eye-catching results reported as statistically significant is consistent with systematic large-scale replication projects in psychology and economics (76–82) where many results failed to replicate. Several factors may contribute to a high false positive risk, such as low statistical power (38–40) and selective reporting of results (42–45). In the hormone and behavior literature, it is easy to see how researcher degrees of freedom might be a problem. Since running experiments involving hormones is more expensive and effortful than running purely behavioral studies, many outcome measures are usually studied, and several control variables are collected. Besides studying main effects, adding interactions with, for example, endogenous hormone levels or 2D:4D also contributes to more tests being performed. There are also some degrees of freedom in defining outcome variables and “outliers.” Previous studies have typically not been based on detailed preanalysis plans describing exact hypotheses and tests which could help limit intentional or unintentional selective reporting of results. The abundance of significant interaction effects in previous studies is also unsurprising, given the small sample sizes and the lack of proper preregistrations. In future work on hormones and economic preferences (or related behaviors) we thus believe that high-powered preregistered studies are the way forward.

## Methods

### Study Location and Sample.

#### Study location.

Data were collected at three locations, Nipissing University’s Social Neuroendocrinology Laboratory in North Bay, ON, Georgian College in Barrie, ON, and Medical Offices in Sudbury, ON, Canada. Participants completed the study in groups of up to seven, in rooms with isolated computer stations.

#### Sample.

Participants were men ranging from 18 to 45 y old. Participants included university students, college students, and community members. Participants were recruited via Facebook, Instagram, Kijiji, posters on campus, booths on campus, classroom announcements, a research participant database, posters in the community, booths in local malls, newspaper ads, radio ads, and word-of-mouth. Data collection began on February 13, 2018, and concluded on December 13, 2023, when we reached our target sample size of 1,000 (the time period of the data collection was substantially increased due to the COVID-19 pandemic).

#### Participant payment.

Participants received $40 for their participation (requiring 2 h of their time), plus a variable bonus depending on their performance on specific tasks, decisions, and chance. They made multiple decisions in each economic preference task and they were randomly paid for one of these decisions in each task (with the dictator game and the charity game counting as one task in terms of the payments). This was explained to participants in the experimental instructions. However, they did not learn about their earnings in any task until after the experiment, preventing earnings in one task from affecting behavior in subsequent tasks. For the dictator and related charity game, participants could potentially get paid as a recipient in addition to the payment as a dictator, if a dictator game where they acted as a recipient was drawn for real payment. This probability was low, however, as only one out of 13 decisions (one dictator game decision and 12 charity game decisions was drawn for real payment). As noted by a reviewer, it is not clear whether participants understood that they could also be paid as a recipient (although it was made clear that if a game where they were the dictator was drawn for real payment, the payment they gave would be given to the other participant).

## Experimental Procedure

Below we describe the experimental procedure in chronological order.

### Informed Consent and Rules.

- Participants read and signed an informed consent form including the rules of the study.

### Trait Measures.

- Ten-Item Personality Inventory (see https://gosling.psy.utexas.edu/scales-weve-developed/ten-item-personality-measure-tipi/ for additional details).
- Self-Report Dominance and Prestige Scale (see https://ubc-emotionlab.ca/research-rtools/dominance-prestige-scales/ for additional details).
- Self-Control Scale (83).
- Barratt Impulsivity Scale (84).
- Thirty-Item Self-Construal Scale (85).

#### Saliva sample one.

Participants provided their first saliva sample by chewing on a synthetic swab for 30 s. Saliva samples were stored at −20 °C until the time of hormone determination, at which point they were thawed, centrifuged, and the supernatant was extracted and analyzed (in duplicate). Salivary testosterone and cortisol were assayed using commercially available enzyme immunoassay kits from DRG International, Inc. Intra- and interassay CVs for testosterone were 9.6% and 12.2%, respectively. Intra- and interassay CVs for cortisol were 9.92% and 7.03%, respectively. After saliva sample one, participants changed into a new T-shirt that they wore during the study.

### Self-Administration of Testosterone or Placebo.

Under the supervision of a research assistant, participants self-administered 11 mg of testosterone or placebo (5.5 mg per nostril) using 1-mm amber syringes. Both the testosterone gel and the placebo gel were prepared by a third-party compounding pharmacy technician. The intranasal testosterone gel (Natesto) used in the current work was developed by Acerus Pharmaceuticals for the treatment of male hypogonadism (https://www.natesto.com/). The pharmacokinetic properties of Natesto were first reported by Rogol and colleagues (86) in an experiment with hypogonadal men. This preparation was found to restore serum testosterone concentrations and to improve erectile function, mood, bone density, and body composition (86). Moreover, this preparation was found to maintain normal levels of FSH, LH, and sperm count (87). A pharmacokinetic study with healthy eugonadal men was performed to examine serum testosterone responses to a single 11 mg dose of testosterone (Natesto). The intranasal testosterone product comes in a dispenser and patients must pump the dispenser in each nostril (delivers 5.5 mg of testosterone per nostril; https://www.natesto.com/how-to-use-natesto/). Because the researchers did not want to share dispensers across participants, a compounding pharmacist prepared individual doses in medical grade syringes. The technician filled syringes with either 0.12 mL of testosterone or placebo. The volume of 0.12 mL was determined to deliver 5.5 mg of testosterone (or placebo) and account for the void space in each syringe. This volume was determined by using the density (weight per volume) of testosterone (or placebo) and then weighing delivered amounts until the correct volume was consistently released. Results from this experiment indicated that a single dose of intranasal testosterone rapidly increased serum testosterone concentrations and that levels achieved were within the high-normal physiological range (47). During data collection (fall of 2019), the testosterone nasal product was recalled and not available to consumers (https://s2.q4cdn.com/417379002/files/doc_downloads/20190807-Natesto-Dear-Health-Care-Professional.pdf). The reason for the recall was that the dispenser malfunctioned for certain lots of the drug, leading to patients receiving lower doses of the drug. Fortunately, we did not use the dispenser and each dose was carefully weighted to ensure that participants received exactly 5.5 mg of testosterone per nostril. Given the uncertainty regarding when the drug would be available to consumers, we decided to use a compounded version of the nasal gel for the remainder of our study (participants 697 to 1,000). However, prior to resumption of data collection we conducted a pharmacokinetic experiment to ensure that the compounded drug would yield a similar serum testosterone response as the brand name drug Natesto. Consistent with previous work using Natesto (see ref. 47), results indicated that a single dose of compounded intranasal testosterone gel (11 mg) rapidly increased serum testosterone concentrations in men (46). Specifically, levels were above baseline 15 min after administration and remained elevated up to 120 min after administration. These results indicated that the compounded intranasal testosterone gel produced similar increases in serum testosterone concentrations compared to Natesto intranasal testosterone gel.

### Soak Period (30 min).

- Participants answered a series of demographic questions.
- Participants completed the MacArthur Scale of Subjective Social Status (see https://www.macses.ucsf.edu/research/psychosocial/subjective.php#measurement for additional details).
- Participants reported their income.
- Participants answered two questions regarding sleep.
- Participants had a picture of their faces taken.
- Participants had their hands scanned.

### Saliva Sample Two.

Participants provided their second saliva sample using the same procedure as before.

### Mouthwash Sample.

Participants provided a mouthwash sample.

### Primary Tasks.

(tasks b–f and h are included as outcome measures in this paper and task a as an independent variable; tasks g, i, and j are not reported in this paper).

- Brief mood introspection scale (see https://www.midss.org/sites/default/files/bmis.pdf for additional details)
- Risk task
- Ultimatum game
- Trust game
- Dictator game
- Charity game
- Cognitive reflection test/confidence task
- Competition task
- Sexual motivation task
- Grip-strength tasks

### Closing Tasks.

- Participants answered two study satisfaction questions.
- Participants answered a few questions regarding the purpose of the study.
- Participants reported whether or not they had participated in a similar study before.
- Participants answered two treatment belief questions.

### Economic Preference Tasks and Primary Outcome Measures.

Below we provide some information about the economic preference tasks used, with more detailed information provided in *SI Appendix*, sections 1–6. We only define the outcome variables and hypotheses tested below for the main hypothesis tests, with more information about exploratory analyses in *SI Appendix*, sections 1–7. The study also included a cognitive reflection test (CRT), a sexual motivation task and a grip strength task as main outcome measures. The CRT is part of an “adversarial collaboration” to be reported in a separate paper, and the two remaining tasks will also be reported in separate papers and have no clear link to economic preferences.

### Risk Task.

In the risk task, participants were asked to make forty choices between two options. For each choice, one option was a lottery with two equally likely outcomes (e.g., $4 or −$1, both with 50% probability) and the other option was a certain outcome (e.g., $3 with 100% probability). The series of choices was tailored to each participant using an adaptive algorithm (56–58). Participants began the task with an endowment of ten dollars, and one of their choices was randomly selected for payment. Bayesian estimates of Risk Aversion (rho) and Loss Aversion (lambda) were estimated for each participant, based on their decisions (56–58). The Risk Aversion and Loss Aversion outcome variables, based on prospect theory (88), are used as outcome measures in the analyses and it is hypothesized that testosterone will decrease both risk aversion and loss aversion based on the correlational results of Apicella et al. (26) and Schipper (29). See *SI Appendix*, section 1 for more details, including detailed information about all the preregistered tests of the risk task data.

### Ultimatum Game.

Participants played the ultimatum game (52) in the role of both proposer and responder after first completing a comprehension check. As a proposer, they could decide how much of $10 to offer to the responder, with the following four discrete choices: $0, $2, $3, and $5. They repeated this decision five times and the average across these five decisions is the Proposer Value outcome variable used in the analyses. As a responder, they decided whether to accept or reject the following five proposer offers in random order: $0, $2, $3, $5, and $5. The Responder Value outcome variable is defined as the fraction of the three unfair offers ($0, $2, $3) that were rejected (the $5 offer implies an equal split where the responder has little reason to reject). The Proposer Value and Responder Value variables are used in the hypothesis tests and it is hypothesized that testosterone will increase both variables based on Eisenegger et al. (13) for Proposer Value and Burnham (25) for Responder Value [although note that Zak et al. (12) reported that testosterone decreased the Proposer Value]. One of the in total 10 rounds as proposer or responder was selected for real payment. See *SI Appendix*, section 2 for more details, including detailed information about all the preregistered tests of the ultimatum game data.

### Trust Game.

Participants played the trust game (53) in the role of both investor and trustee after first completing a comprehension check. As a proposer, they could decide how much of $10 to send to the responder, with the following four discrete choices: $0, $2, $6, and $10. They repeated this decision five times and the average across these five decisions is the Investor Value outcome variable used in the analyses. As a trustee, they decided how much of the tripled amount to return to the investor for the following five investments in random order: $2, $6, $6, $10, and $10. The Trustee Value outcome variable is defined as the average fraction of the tripled investment that is returned to the investor. The Investor Value and Trustee Value variables are used in the hypotheses tests and it is hypothesized that testosterone will decrease Investor Value and increase Trustee Value based on Boksem et al. (16). One of the total 10 rounds as investor or trustee was selected for real payment. See *SI Appendix*, section 3 for more details, including detailed information about all the preregistered tests of the trust game data.

### Dictator Game.

Participants played the dictator game (54) in the role of dictator after first completing a comprehension check. They decided how much of $10 to give to another participant (the recipient) with the following 11 discrete choices: $0, $1, $2, $3, $4, $5, $6, $7, $8, $9 and $10. The decision was made once and the amount given is the Dictator Value outcome variable used in the analyses and it is hypothesized that testosterone will decrease this value based on Zak et al. (12) that reported that testosterone decreased generosity (measured as the difference between offers and rejection thresholds in the ultimatum game). See *SI Appendix*, section 4 for more details, including detailed information about all the preregistered tests of the dictator game data.

### Charity Game.

Participants played twelve rounds of what we refer to as the “charity game” (60, 61), which can be viewed as a version of the dictator game. In each round of this game, participants divided an endowment of $6, $8, or $10 between themselves and North Bay Food Bank (a charity that works within the community to gather and distribute food to those in need), in increments of $1. A multiplier was assigned for each round, such that North Bay Food Bank would receive $0.75, $1.00, $1.25, or $1.75 for every dollar donated. Participants played a total of 12 rounds of this game (3 endowments × 4 multipliers) in a random order. The average percentage donation in each round is the Percentage Donated outcome variable used in the analyses. It is hypothesized that testosterone will affect the relationship between the Price of Donating (the inverse of the multiplier) and testosterone treatment on the Percentage Donated, with a hypothesized positive interaction effect (implying that testosterone decreases the sensitivity to the Price of Donating). This hypothesis is motivated by the hypothesis that testosterone increases status-seeking behavior (7) and the “competitive altruism hypothesis” postulating that the status of altruistic behavior increases if it is more costly (59). One of the 12 charity game decisions or the dictator game decision was randomly selected for real payment. See *SI Appendix*, section 5 for more details, including detailed information about all the preregistered tests of the charity game data.

### Competition Task.

This task (55) consisted of three parts and one of these parts was randomly selected for payment. In each part, participants were given 2 min to solve as many math problems as possible. Each problem entailed adding together four two-digit numbers (e.g., 63 + 19 + 12 + 44 = …). Thirty-five problems were presented in each part and participants were provided with a pen and paper. In the first part, participants earned $0.50 for each problem that they solved correctly. In the second part, they earned $0.75 for each problem that they solved correctly if they solved more problems than an anonymous competitor, and $0.00 if they solved fewer problems correctly than their anonymous competitor (if there was a tie they earned $0.50 for each problem that they solved correctly). In the third part, they had to choose between a piece rate payment scheme ($0.50 for each problem that they solved correctly) and a competitive payment scheme ($0.75 per problem solved correctly if they solved more problems than their competitor solved in part two, $0.00 if they solved fewer, and $0.50 for each problem that they solve correctly in case of a tie). The choice in the third part is the Willingness to Compete (1 = choosing the competitive payment scheme) outcome variable used in the analyses. The Willingness to Compete variable is used in the hypothesis tests and it is hypothesized that testosterone will increase the Willingness to Compete, in line with the correlational result in ref. 31. See *SI Appendix*, section 6 for more details, including detailed information about all the preregistered tests of the competition task data.

### Deviations from Preregistered Tests.

There was some ambiguity in the preanalysis plan and it did not specify if the *t* tests used in the primary hypotheses tests would be based on assuming equal variances or not or if robust SE would be estimated for the ordinary least squares regressions used in many of the robustness tests and exploratory analyses. All the *t* tests were based on assuming equal variances and all ordinary least squares regressions were estimated with robust SE. There were also some changes in the definitions of the independent variables included in the regression analyses, detailed in *SI Appendix*, section 9, necessitated by the fact that some ambiguity remained in the preregistration or that these variables were not collected or coded in the raw data exactly as preregistered. There was also an error in the preregistration for the hypothesized direction of the robustness tests and exploratory analyses of the primary outcome variable Risk Aversion. The preregistration correctly reported the expected direction of the hypotheses verbally, but the predicted signs in the regressions were incorrect and inconsistent with the verbal descriptions as a higher risk aversion parameter (rho) implies less risk aversion (and the predicted signs were based on that a higher risk aversion parameter implies more risk aversion). For the charity game analysis the preregistered linear mixed-effect model also failed to converge in three exploratory analyses and the robustness tests of these analyses, and were replaced by an alternative regression model (see *SI Appendix*, section 5 for more details). As noted above we also added a not-preregistered exploratory analysis of financial risk taking assessed on a 0 to 10 scale from completely unwilling (0) to completely willing (10) (56, 65), and four sets of non-preregistered robustness tests and exploratory analyses suggested by reviewers.

### Ethical Approval.

The study was approved by the Nipissing University Research Ethics Board (Protocol #101652).

## Supplementary Material

Appendix 01 (PDF)

## Data Availability

The data reported in this paper and the analysis scripts generating all results in the main text and *SI Appendix* are available at the project’s OSF repository (https://osf.io/6yrgf/) (89).
