# Supplementary material for: Investigating the effects of single-dose intranasal testosterone on economic preferences in a large randomized trial of men
Source: Proc Natl Acad Sci U S A. 2025 Sep 23;122(39):e2508519122. doi: 10.1073/pnas.2508519122 (PMC12501127; doi:10.1073/pnas.2508519122)
Supplement: Supplementary file 1 — Appendix 01 (PDF) [file pnas.2508519122.sapp.pdf]

Supporting Information

Investigating the effects of single-dose intranasal testosterone on economic preferences in a large randomized trial of men

Anna Dreber[1,2,*], Magnus Johannesson[1], Gidi Nave[3], Coren Apicella[4], Shawn Geniole[5], Taisuke Imai[6], Erik Knight[7], Dylan Manfredi[3], Pranjal H. Mehta[8], Valentina Proietti[4], Steven J. Stanton[9], Alina Zeltikova[10], Francesca Luberti[11], Triana Ortiz[11], Justin M. Carré[11*]

[1]Department of Economics, Stockholm School of Economics, Stockholm, Sweden. [2]Department of Economics, University of Innsbruck, Innsbruck, Austria. [3]Marketing Department, Wharton School, University of Pennsylvania, Philadelphia, USA. [4]Department of Psychology, University of Pennsylvania, Philadelphia, USA. [5]Department of Psychology, University of the Fraser Valley, Abbotsford, Canada. [6]Institute of Social and Economic Research, The University of Osaka, Osaka, Japan. [7]Department of Psychology and Neuroscience, University of Colorado Boulder, Boulder, USA. [8]Department of Experimental Psychology, University College London, London, UK. [9]Department of Management and Marketing, School of Business Administration, Oakland University, Rochester, USA. [10]Department of Economics, University College London, London, UK.[11]Department of Psychology, Nipissing University, North Bay, Canada

[*] To whom correspondence should be addressed:

Anna Dreber
Department of Economics, Stockholm School of Economics
Box 6501, SE-113 83 Stockholm, Sweden.
Email: anna.dreber@hhs.se

and

Justin M. Carré
Department of Psychology, Nipissing University
100 College Dr. North Bay, ON P1B8L7, Canada.
Email: justinca@nipissingu.ca

This PDF file includes:

SI Methods
SI References
Tables S1-S29

SI Methods

Below we provide further details on the different economic preference tasks and outcome measures and the preregistered hypothesis tests and exploratory analyses (sections 1-6), the added non-preregistered analyses (sections 7-8), and the definition of the independent variables used in the analyses (section 9). The descriptions of the tasks and the hypotheses and tests below are taken from the posted pre-analysis plans for each task. We use an independent-samples t-test in most primary hypothesis tests but the pre-analysis plan did not specify if these t-tests would be based on assuming equal variance across the two groups or not; but all these tests are carried out assuming equal variances (which is the standard version of the independent samples t-test). The pre-analysis plan did also not specify if the ordinary least squares regression models would be estimated with or without robust standard errors, but all the ordinary least squares regressions are estimated with robust standard errors which is standard in economics (but robust standard errors are not used for the linear mixed-effect model regressions or the logistic and ordered logistic regressions models).

Some robustness tests and exploratory analyses use an ordered logistic regression for outcome variables with a fixed number of ordered decisions and some of these analyses use logistic regression for binary outcomes; but any tests involving an interaction coefficient are based on ordinary least squares regression or a linear mixed effect model as interactions are difficult to interpret in non-linear models such as logistic regression (1). We follow the pre-registered regression models in the analyses and tests, although the choice of regression model is not always consistent across the different tasks as separate pre-registrations were filed for each task. For the charity game task we preregistered to use a linear mixed-effect model, but this model did not converge for three exploratory analyses and the robustness tests of these exploratory analyses and were therefore replaced by ordinary least squares regressions with clustering of standard errors on the participant level (for the main hypothesis and the robustness test of this hypothesis tests the linear mixed-effect models converge and we report these results, but for these results we also checked the similarity in results for an ordinary least squares regression with clustering on the participant level and the results are almost identical; see more on this below).

1. Risk task

1.1 Procedure and outcome measures

Procedure. Participants were asked to make forty choices between two options. For each choice, one option was a lottery with two equally-likely outcomes (e.g. \$4 or -\$1, both with 50% probability) and the other option was a certain outcome (e.g. \$3 with 100% probability). Participants began the task with an endowment of ten dollars, and one of their choices was randomly selected for payment. The series of choices was tailored to each participant using an adaptive algorithm, Dynamically Optimized Sequential Experimentation (DOSE) (2). The method has been applied in several studies estimating loss aversion, including those by Chumbley et al. (3), Krajbich et al. (4), and Chapman et al. (5).

Data transformations. Participants' behavior is modeled according to Prospect Theory (6), and based on the 40 choices the risk aversion parameter (rho) and the loss aversion parameter (lambda) of prospect theory are estimated. The method generates Bayesian posteriors over the hypotheses (combinations of model parameters) after each question. We construct "parameter estimates" for each participant at each stage of the task using the posterior mean and use the posterior after the last choice as the estimate of risk aversion (rho) and loss aversion (lambda).

1.2 Pre-registered hypothesis tests and exploratory analyses

Hypothesis 1: Participants will exhibit less risk aversion after testosterone administration than after placebo administration. An independent-samples t-test will be used to test this hypothesis. Our threshold for statistical significance will be alpha = 0.005, whereas our threshold for suggestive evidence will be alpha = 0.05.

Hypothesis 2: Participants will exhibit less loss aversion after testosterone administration than after placebo administration. An independent-samples t-test will be used to test this hypothesis. Our threshold for statistical significance will be alpha = 0.005, whereas our threshold for suggestive evidence will be alpha = 0.05.

Note that the predicted signs below for the regression coefficients in the robustness checks and the exploratory analyses have been reversed compared to the preregistration for all tests involving risk aversion. The preregistration correctly reports the expected signs verbally (the first sentence below in each robustness check and each exploratory analysis involving risk aversion); but the predicted signs in the regressions are incorrect and inconsistent with the verbal descriptions in the preregistration as a higher risk aversion parameter (rho) implies less risk aversion. The predictions in the preregistration for loss aversion are correct as higher loss aversion (lambda) parameter implies higher loss aversion.

Robustness Check 1: Participants will exhibit less risk aversion after testosterone administration than after placebo administration, when controlling for right-hand 2D:4D, pleasant-unpleasant mood, arousal-calm mood, time of day, and site. This is tested in the following regression model:

Regression model: Ordinary least squares.

Dependent variable: Risk Aversion.

Independent variables: Treatment (Testosterone = 1, Placebo = 0), Right-Hand 2D:4D, Pleasant-Unpleasant mood, Arousal-Calm Mood, Time of Day, Site.

Hypothesis: Positive coefficient of Treatment.

Robustness Check 2: Participants will exhibit less loss aversion after testosterone administration than after placebo administration, when controlling for right-hand 2D:4D, pleasant-unpleasant mood, arousal-calm mood, time of day, and site. This is tested in the following regression model:

Regression model: Ordinary least squares.

Dependent variable: Loss Aversion.

Independent variables: Treatment (Testosterone = 1, Placebo = 0), Right-Hand 2D:4D, Pleasant-Unpleasant mood, Arousal-Calm Mood, Time of Day, Site.

Hypothesis: Negative coefficient of Treatment.

Exploratory Hypothesis 1: The association between treatment and risk aversion will be stronger for participants with greater self-construal. This is tested in the following regression model:

Regression model: Ordinary least squares.

Dependent variable: Risk Aversion.

Independent variables: Treatment (Testosterone = 1, Placebo = 0), Self-Construal, Treatment*Self-Construal.

Hypothesis: Positive coefficient of interaction Treatment*Self-Construal.

Exploratory Hypothesis 2: The association between treatment and risk aversion will be stronger for participants with lower basal cortisol. This is tested in the following regression model:

Regression model: Ordinary least squares.

Dependent variable: Risk Aversion.

Independent variables: Treatment (Testosterone = 1, Placebo = 0), Basal Cortisol, Treatment*Basal Cortisol.

Hypothesis: Negative coefficient of interaction Treatment*Basal Cortisol.

Exploratory Hypothesis 3: Participants with higher basal testosterone will exhibit less risk aversion. This is tested in the following regression model:

Regression model: Ordinary least squares.

Dependent variable: Risk Aversion.

Independent variables: Treatment (Testosterone = 1, Placebo = 0), Basal Testosterone.

Hypothesis: Positive coefficient of Basal Testosterone.

Exploratory Hypothesis 4: Participants with higher basal testosterone will exhibit less loss aversion. This is tested in the following regression model:

Regression model: Ordinary least squares.

Dependent variable: Loss Aversion.

Independent variables: Treatment (Testosterone = 1, Placebo = 0), Basal Testosterone.

Hypothesis: Negative coefficient of Basal Testosterone.

Exploratory Hypothesis 5: Participants with higher basal cortisol will exhibit greater risk aversion. This is tested in the following regression model:

Regression model: Ordinary least squares.

Dependent variable: Risk Aversion.

Independent variables: Treatment (Testosterone = 1, Placebo = 0), Basal Cortisol.

Hypothesis: Negative coefficient of Basal Cortisol.

Exploratory Hypothesis 6: Participants with higher basal cortisol will exhibit greater loss aversion. This is tested in the following regression model:

Regression model: Ordinary least squares.

Dependent variable: Loss Aversion.

Independent variables: Treatment (Testosterone = 1, Placebo = 0), Basal Cortisol.

Hypothesis: Positive coefficient of Basal Cortisol.

Exploratory Hypothesis 7: The association between basal testosterone and risk aversion will be stronger for participants with lower basal cortisol. This is tested in the following regression model:

Regression model: Ordinary least squares.

Dependent variable: Risk Aversion.

Independent variables: Treatment (Testosterone = 1, Placebo = 0), Basal Testosterone, Basal Cortisol, Basal Testosterone*Basal Cortisol.

Hypothesis: Negative coefficient of interaction Basal Testosterone*Basal Cortisol.

Exploratory Hypothesis 8: The association between basal testosterone and loss aversion will be stronger for participants with lower basal cortisol. This is tested in the following regression model:

Regression model: Ordinary least squares.

Dependent variable: Loss Aversion.

Independent variables: Treatment (Testosterone = 1, Placebo = 0), Basal Testosterone, Basal Cortisol, Basal Testosterone*Basal Cortisol.

Hypothesis: Positive coefficient of interaction Basal Testosterone*Basal Cortisol.

Exploratory Hypothesis 9: The association between treatment and loss aversion will be stronger for participants with lower basal cortisol. This is tested in the following regression model:

Regression model: Ordinary least squares.

Dependent variable: Loss Aversion.

Independent variables: Treatment (Testosterone = 1, Placebo = 0), Basal Cortisol, Treatment*Basal Cortisol.

Hypothesis: Positive coefficient of interaction Treatment*Basal Cortisol.

2. The ultimatum game

2.1 Procedure and outcome measures

Procedure. Participants started by reading the instructions for part one of the ultimatum game, which appeared as follows:

"Now, you will play five rounds of this game in the role of a "proposer". In each round, you will be paired with a different "responder" and receive $10. Your job is to decide how to divide the $10 between yourself and the "responder". You can choose to do one of the following:

a. Keep $10 and give the "responder" $0
b. Keep $8 and give the "responder" $2
c. Keep $7 and give the "responder" $3
d. Keep $5 and give the "responder" $5

Your decision will be referred to as your offer. The “responder” can choose to accept or reject your offer. If the “responder” accepts your offer, then both of you will receive money according to your offer. If the “responder” rejects your offer, then both of you will receive nothing.”

After reading the instructions, participants completed a 4-item task comprehension questionnaire to verify that they fully understood all task dynamics. Upon completion of the comprehension questionnaire, participants immediately began part one of the ultimatum game. They responded to the following scenario five times:

“How do you offer to divide the $10 between you and the “responder”?

a. $10 for me, $0 for the “responder”
b. $8 for me, $2 for the “responder”
c. $7 for me, $3 for the “responder”
d. D. $5 for me, $5 for the “responder”

After responding to the preceding scenario five times, participants read instructions for part two of the ultimatum game, which appeared as follows.

“Now, you will play five rounds of this game in the role of a “responder”. In each round, you will be paired with a different “proposer” and be shown the “proposer's” offer. Your job is to accept or reject the “proposer’s” offer. If you accept the “proposer’s” offer, both of you will receive money according to the “proposer’s” offer. If you reject the “proposer's” offer, both of you will receive nothing.”

After reading the instructions for part two of the ultimatum game, participants immediately began part two of the ultimatum game. They responded to the following five scenarios, in a random order.

1. The “proposer” has offered to give you $0 and keep $10. Do you accept or reject the “proposer’s” offer?

a. I accept the “proposer's” offer (you will receive $0 and the “proposer” will receive $10).
b. I reject the “proposer's” offer (you will receive $0 and the “proposer” will receive $0).

2. The “proposer” has offered to give you $2 and keep $8. Do you accept or reject the “proposer’s” offer?

a. I accept the “proposer's” offer (you will receive $2 and the “proposer” will receive $8).
b. I reject the “proposer's” offer (you will receive $0 and the “proposer” will receive $0).

3. The “proposer” has offered to give you $3 and keep $7. Do you accept or reject the “proposer’s” offer?

   a. I accept the “proposer's” offer (you will receive $3 and the “proposer” will receive $7).
   b. I reject the “proposer's” offer (you will receive $0 and the “proposer” will receive $0).

4. The “proposer” has offered to give you $5 and keep $5. Do you accept or reject the “proposer’s” offer?
   a. I accept the “proposer's” offer (you will receive $5 and the “proposer” will receive $5).
   b. I reject the “proposer's” offer (you will receive $0 and the “proposer” will receive $0).

5. The “proposer” has offered to give you $5 and keep $5. Do you accept or reject the “proposer’s” offer?
   a. I accept the “proposer's” offer (you will receive $5 and the “proposer” will receive $5).
   b. I reject the “proposer’s” offer (you will receive $0 and the “proposer” will receive $0).

Payment. Participants played a total of ten rounds of the ultimatum game (five rounds in part one and five rounds in part two). One of these ten rounds was randomly selected for payment, in accordance with the following procedure. Each participant was randomly assigned to the role of a proposer or a responder, such that half of the participants were proposers and half of the participants were responders. Then, each proposer was paired with a responder. Then, one of each proposer’s offers was randomly selected. Their offer was paired with their partner’s corresponding response to this offer for the sake of payment determination. Participants did not receive immediate feedback about their partner’s decisions or their payment. They were paid approximately one month after they completed the study.

Data Transformations. First, a Proposer Value was calculated for each participant in accordance with the choices that they made in part one of the ultimatum game, as shown below.

   a. Proposer Value=0
   b. Proposer Value=2
   c. Proposer Value=3
   d. Proposer Value=5

Thereafter the Proposer Values for each of the five times that participants responded to the scenario in part one of the ultimatum game was averaged to yield a variable denoted Proposer Value.

The Responder Value was calculated by determining the number of times that participants chose option b for scenarios one through three in part two of the ultimatum game and dividing this number by three.

2.2 Pre-registered hypothesis tests and exploratory analyses

Hypothesis 1: We predict that participants' average Proposer Value will be higher after testosterone administration than after placebo administration. We will test this hypothesis by conducting a two-sample t-test comparing the average Proposer Value in the testosterone group to the average Proposer Value in the placebo group. Our threshold for statistical significance will be alpha = 0.005, whereas our threshold for suggestive evidence will be alpha = 0.05.

Hypothesis 2: We predict that participants' average Responder Value will be higher after testosterone administration than after placebo administration. We will test this hypothesis by conducting a two-sample t-test comparing the average Responder Value in the testosterone group to the average Responder Value in the placebo group. Our threshold for statistical significance will be alpha = 0.005, whereas our threshold for suggestive evidence will be alpha = 0.05.

Robustness Check 1: We predict that Proposer Values will be higher after testosterone administration than after placebo administration when controlling for right-hand 2D:4D, pleasant-unpleasant mood, arousal-calm mood, time of day, and site. This is tested in the following regression model:

Regression model: Ordinary least squares.

Dependent variable: Proposer Value.

Independent variables: Treatment (Testosterone = 1, Placebo = 0), Right-Hand 2D:4D, Pleasant-Unpleasant mood, Arousal-Calm Mood, Time of Day, Site.

Hypothesis: Positive coefficient of Treatment.

Robustness Check 2: We predict that Proposer Values will be higher after testosterone administration than after placebo administration when controlling for right-hand 2D:4D, pleasant-unpleasant mood, arousal-calm mood, time of day, site, and treatment belief. This is tested in the following regression model:

Regression model: Ordinary least squares.

Dependent variable: Proposer Value.

Independent variables: Treatment (Testosterone = 1, Placebo = 0), Right-Hand 2D:4D, Pleasant-Unpleasant mood, Arousal-Calm Mood, Time of Day, Site, Treatment Belief.

Hypothesis: Positive coefficient of Treatment.

Robustness Check 3: We predict that Responder Values will be higher after testosterone administration than after placebo administration when controlling for right-hand 2D:4D, pleasant-unpleasant mood, arousal-calm mood, time of day, and site. This is tested in the following regression model:

Regression model: Ordered logistic regression.

Dependent variable: Responder Value.

Independent variables: Treatment (Testosterone = 1, Placebo = 0), Right-Hand 2D:4D, Pleasant-Unpleasant mood, Arousal-Calm Mood, Time of Day, Site.

Hypothesis: Positive coefficient of Treatment.

Robustness Check 4: We predict that Responder Values will be higher after testosterone administration than after placebo administration when controlling for right-hand 2D:4D, pleasant-unpleasant mood, arousal-calm mood, time of day, site, and treatment belief. This is tested in the following regression model:

Regression model: Ordered logistic regression.

Dependent variable: Responder Value.

Independent variables: Treatment (Testosterone = 1, Placebo = 0), Right-Hand 2D:4D, Pleasant-Unpleasant mood, Arousal-Calm Mood, Time of Day, Site, Treatment Belief.

Hypothesis: Positive coefficient of Treatment.

Exploratory Hypothesis 1: Participants with higher basal testosterone will exhibit higher Proposer Values. This is tested in the following regression model:

Regression model: Ordinary least squares.

Dependent variable: Proposer Value.

Independent variables: Treatment (Testosterone = 1, Placebo = 0), Basal Testosterone.

Hypothesis: Positive coefficient of Basal Testosterone.

Exploratory Hypothesis 2: The association between basal testosterone and Proposer Values will be stronger for participants with lower basal cortisol. This is tested in the following regression model:

Regression model: Ordinary least squares.

Dependent variable: Proposer Value.

Independent variables: Treatment (Testosterone = 1, Placebo = 0), Basal Testosterone, Basal Cortisol, Basal Testosterone*Basal Cortisol.

Hypothesis: Negative coefficient of Basal Testosterone*Basal Cortisol.

Exploratory Hypothesis 3: Participants with higher basal testosterone will exhibit higher Responder Values. This is tested in the following regression model:

Regression model: Ordered logistic regression.

Dependent variable: Responder Value.

Independent variables: Treatment (Testosterone = 1, Placebo = 0), Basal Testosterone.

Hypothesis: Positive coefficient of Basal Testosterone.

Exploratory Hypothesis 4: The association between basal testosterone and Responder Values will be stronger for participants with lower basal cortisol. This is tested in the following regression model:

Regression model: Ordinary least squares.

Dependent variable: Responder Value.

Independent variables: Treatment (Testosterone = 1, Placebo = 0), Basal Testosterone, Basal Cortisol, Basal Testosterone*Basal Cortisol.

Hypothesis: Negative coefficient of interaction Basal Testosterone*Basal Cortisol.

Exploratory Hypothesis 5: The association between treatment and Responder Values will be stronger for participants with lower basal cortisol. This is tested in the following regression model:

Regression model: Ordinary least squares.

Dependent variable: Responder Value.

Independent variables: Treatment (Testosterone = 1, Placebo = 0), Basal Cortisol, Treatment*Basal Cortisol.

Hypothesis: Negative coefficient of interaction Treatment*Basal Cortisol.

3. The trust game

3.1 Procedure and outcome measures

Procedure. Participants started by reading the instructions for part one of the trust game, which appeared as follows:

"In each of the following five rounds of this game, you will be paired with another participant and receive $10. Your job is to decide how much of this $10 you want to keep and how much you want to send to the other participant. You can choose to do one of the following:

a. Keep $10 and send $0 to the other participant
b. Keep $8 and send $2 to the other participant
c. Keep $4 and send $6 to the other participant
d. Keep $0 and send $10 to the other participant

The amount that you decide to send to the other participant will be tripled (i.e. for every $1 you decide to send, the other participant will receive $3). The other participant will decide how much of this tripled amount to return to you. You will earn whatever money is returned to you plus the share of the initial $10 that you decided to keep. The other participant will receive the share of the tripled amount that they do not return to you."

After reading the instructions, participants completed a 2-item task comprehension questionnaire to verify that they fully understood all task dynamics. Upon completion of the comprehension questionnaire, participants immediately began part one of the trust game. They responded to the following scenario five times:

“You have $10. How much money do you want to keep? How much money do you want to send to the other participant?

a. I want to keep $10. I want to send $0 to the other participant.
b. I want to keep $8. I want to send $2 to the other participant.
c. I want to keep $4. I want to send $6 to the other participant.
d. I want to keep $0. I want to send $10 to the other participant.

After responding to the preceding scenario five times, participants read instructions for part two of the trust game, which appeared as follows:

“Now you will play five rounds of this game in the other role. In each round, you will receive a quantity of money equal to three times the amount that another participant decided to send to you. Your job is to decide how much of this tripled amount you want to keep and how much of this tripled amount you want to return to the other participant. You will receive the amount of money that you decide to keep. The other participant will receive the amount of money that you decide to return to them (plus the share of the initial $10 that they decided to keep).”

After reading the instructions for part two of the trust game, participants immediately began part two of the trust game. They responded to the following five scenarios, in a random order.

1. The other participant has sent $2 to you. This amount tripled equals $6. How much of this money do you want to keep? How much of this money do you want to return to the other participant?

a. I want to keep $6. I want to return $0 to the other participant.
b. I want to keep $5. I want to return $1 to the other participant.
c. I want to keep $4. I want to return $2 to the other participant.
d. I want to keep $3. I want to return $3 to the other participant.
e. I want to keep $2. I want to return $4 to the other participant.
f. I want to keep $1. I want to return $5 to the other participant.
g. I want to keep $0. I want to return $6 to the other participant.

2. The other participant has sent $6 to you. This amount tripled equals $18. How much of this money do you want to keep? How much of this money do you want to return to the other participant?

a. I want to keep $18. I want to return $0 to the other participant.
b. I want to keep $17. I want to return $1 to the other participant.
c. I want to keep $16. I want to return $2 to the other participant.
d. I want to keep $15. I want to return $3 to the other participant.
e. I want to keep $14. I want to return $4 to the other participant.
f. I want to keep $13. I want to return $5 to the other participant.
g. I want to keep $12. I want to return $6 to the other participant.
h. I want to keep $11. I want to return $7 to the other participant.
i. I want to keep $10. I want to return $8 to the other participant.
j. I want to keep $9. I want to return $9 to the other participant.
k. I want to keep $8. I want to return $10 to the other participant.
l. I want to keep $7. I want to return $11 to the other participant.
m. I want to keep $6. I want to return $12 to the other participant.
n. I want to keep $5. I want to return $13 to the other participant.
o. I want to keep $4. I want to return $14 to the other participant.
p. I want to keep $3. I want to return $15 to the other participant.
q. I want to keep $2. I want to return $16 to the other participant.
r. I want to keep $1. I want to return $17 to the other participant.
s. I want to keep $0. I want to return $18 to the other participant.

3. The other participant has sent $6 to you. This amount tripled equals $18. How much of this money do you want to keep? How much of this money do you want to return to the other participant?

a. I want to keep $18. I want to return $0 to the other participant.
b. I want to keep $17 I want to return $1 to the other participant.
c. I want to keep $16. I want to return $2 to the other participant.
d. I want to keep $15. I want to return $3 to the other participant.
e. I want to keep $14. I want to return $4 to the other participant.
f. I want to keep $13. I want to return $5 to the other participant.
g. I want to keep $12. I want to return $6 to the other participant.
h. I want to keep $11. I want to return $7 to the other participant.
i. I want to keep $10. I want to return $8 to the other participant.
j. I want to keep $9. I want to return $9 to the other participant.

k. I want to keep $8. I want to return $10 to the other participant.
l. I want to keep $7. I want to return $11 to the other participant.
m. I want to keep $6. I want to return $12 to the other participant.
n. I want to keep $5. I want to return $13 to the other participant.
o. I want to keep $4. I want to return $14 to the other participant.
p. I want to keep $3. I want to return $15 to the other participant.
q. I want to keep $2. I want to return $16 to the other participant.
r. I want to keep $1. I want to return $17 to the other participant.
s. I want to keep $0. I want to return $18 to the other participant.

4. The other participant has sent $10 to you. This amount tripled equals $30. How much of this money do you want to keep? How much of this money do you want to return to the other participant?

a. I want to keep $30. I want to return $0 to the other participant.
b. I want to keep $29. I want to return $1 to the other participant.
c. I want to keep $28. I want to return $2 to the other participant.
d. I want to keep $27. I want to return $3 to the other participant.
e. I want to keep $26. I want to return $4 to the other participant.
f. I want to keep $25. I want to return $5 to the other participant.
g. I want to keep $24. I want to return $6 to the other participant.
h. I want to keep $23. I want to return $7 to the other participant.
i. I want to keep $22. I want to return $8 to the other participant.
j. I want to keep $21. I want to return $9 to the other participant.
k. I want to keep $20. I want to return $10 to the other participant.
l. I want to keep $19. I want to return $11 to the other participant.
m. I want to keep $18. I want to return $12 to the other participant.
n. I want to keep $17. I want to return $13 to the other participant.
o. I want to keep $16. I want to return $14 to the other participant.
p. I want to keep $15. I want to return $15 to the other participant.
q. I want to keep $14. I want to return $16 to the other participant.
r. I want to keep $13. I want to return $17 to the other participant.
s. I want to keep $12. I want to return $18 to the other participant.
t. I want to keep $11. I want to return $19 to the other participant.
u. I want to keep $10. I want to return $20 to the other participant.
v. I want to keep $9. I want to return $21 to the other participant.
w. I want to keep $8. I want to return $22 to the other participant.

x. I want to keep $7. I want to return $23 to the other participant.
y. I want to keep $6. I want to return $24 to the other participant.
z. I want to keep $5. I want to return $25 to the other participant.
aa. I want to keep $4. I want to return $26 to the other participant.
bb. I want to keep $3. I want to return $27 to the other participant.
cc. I want to keep $2. I want to return $28 to the other participant.
dd. I want to keep $1. I want to return $29 to the other participant.
ee. I want to keep $0. I want to return $30 to the other participant.

5. The other participant has sent $10 to you. This amount tripled equals $30. How much of this money do you want to keep? How much of this money do you want to return to the other participant?

a. I want to keep $30. I want to return $0 to the other participant.
b. I want to keep $29. I want to return $1 to the other participant.
c. I want to keep $28. I want to return $2 to the other participant.
d. I want to keep $27. I want to return $3 to the other participant.
e. I want to keep $26. I want to return $4 to the other participant.
f. I want to keep $25. I want to return $5 to the other participant.
g. I want to keep $24. I want to return $6 to the other participant.
h. I want to keep $23. I want to return $7 to the other participant.
i. I want to keep $22. I want to return $8 to the other participant.
j. I want to keep $21. I want to return $9 to the other participant.
k. I want to keep $20. I want to return $10 to the other participant.
l. I want to keep $19. I want to return $11 to the other participant.
m. I want to keep $18. I want to return $12 to the other participant.
n. I want to keep $17. I want to return $13 to the other participant.
o. I want to keep $16. I want to return $14 to the other participant.
p. I want to keep $15. I want to return $15 to the other participant.
q. I want to keep $14. I want to return $16 to the other participant.
r. I want to keep $13. I want to return $17 to the other participant.
s. I want to keep $12. I want to return $18 to the other participant.
t. I want to keep $11. I want to return $19 to the other participant.
u. I want to keep $10. I want to return $20 to the other participant.
v. I want to keep $9. I want to return $21 to the other participant.
w. I want to keep $8. I want to return $22 to the other participant.
x. I want to keep $7. I want to return $23 to the other participant.

y. I want to keep $6. I want to return $24 to the other participant.
z. I want to keep $5. I want to return $25 to the other participant.
aa. I want to keep $4. I want to return $26 to the other participant.
bb. I want to keep $3. I want to return $27 to the other participant.
cc. I want to keep $2. I want to return $28 to the other participant.
dd. I want to keep $1. I want to return $29 to the other participant.
ee. I want to keep $0. I want to return $30 to the other participant.

Payment. Participants played a total of ten rounds of the trust game (five rounds in part one and five rounds in part two). One of these ten rounds was randomly selected for payment, in accordance with the following procedure. Each participant was randomly assigned to the role of an investor or a trustee, such that half of the participants were investors and half of the participants were trustees. Then, each investor was paired with a trustee. Then, one of each investor's investments was randomly selected. Their investment was paired with one of their partner's corresponding responses to this investment for the sake of payment determination. Participants did not receive immediate feedback about their partner's decisions or their payment. They were paid approximately one month after they completed the study.

Data Transformations. First, an Investor Value was calculated for each participant in accordance with the choices that they made in part one of the trust game, as shown below.

a. Investor Value=0
b. Investor Value=2
c. Investor Value=6
d. Investor Value=10

Thereafter the Investor Values for each of the five times that participants respond to the scenario in part one of the trust game was averaged to yield a variable denoted as the Investor Value.

Then, a Trustee Value was calculated for each participant as the fraction of each tripled investment that they decided to return to the investor in part two of the trust game. The Trustee Values for each of the five times that participants responded to the scenarios in part two of the trust game was averaged to yield a variable denoted as the Trustee Value.

3.2 Pre-registered hypothesis tests and exploratory analyses

Hypothesis 1: We predict that participants' average Investor Value will be lower after testosterone administration than after placebo administration. We will test this hypothesis by conducting a two-sample t-test comparing the average Investor Value in the testosterone group

to the average Investor Value in the placebo group. Our threshold for statistical significance will be alpha = 0.005, whereas our threshold for suggestive evidence will be alpha = 0.05.

Hypothesis 2: We predict that participants' average Trustee Value will be higher after testosterone administration than after placebo administration. We will test this hypothesis by conducting a two-sample t-test comparing the average Trustee Value in the testosterone group to the average Trustee Value in the placebo group. Our threshold for statistical significance will be alpha = 0.005, whereas our threshold for suggestive evidence will be alpha = 0.05.

Robustness Check 1: We predict that Investor Values will be lower after testosterone administration than after placebo administration when controlling for risk aversion, right-hand 2D:4D, pleasant-unpleasant mood, arousal-calm mood, time of day, and site. This is tested in the following regression model:

Regression model: Ordinary least squares.

Dependent variable: Investor Value.

Independent variables: Treatment (Testosterone = 1, Placebo = 0), Risk Aversion, Right-Hand 2D:4D, Pleasant-Unpleasant mood, Arousal-Calm Mood, Time of Day, Site.

Hypothesis: Negative coefficient of Treatment.

Robustness Check 2: We predict that Trustee Values will be greater after testosterone administration than after placebo administration when controlling for right-hand 2D:4D, pleasant-unpleasant mood, arousal-calm mood, time of day, and site. This is tested in the following regression model:

Regression model: Ordinary least squares.

Dependent variable: Trustee Value.

Independent variables: Treatment (Testosterone = 1, Placebo = 0), Right-Hand 2D:4D, Pleasant-Unpleasant mood, Arousal-Calm Mood, Time of Day, Site.

Hypothesis: Positive coefficient of Treatment.

Exploratory Hypothesis 1: The association between treatment and Trustee Values will be stronger for higher investment amounts. This is tested in the following regression model:

Regression model: Linear mixed-effect model with five observations per participant (Treatment and Treatment*Investment Amount treated as fixed effects; Investment Amount and its intercept treated as random effects).

Dependent variable: Trustee Value.

Independent variables: Treatment (Testosterone = 1, Placebo = 0), Investment Amount, Treatment*Investment Amount.

Hypothesis: Positive coefficient of interaction Treatment*Investment Amount.

Exploratory Hypothesis 2: Participants with higher basal testosterone will exhibit lower Investor Values. This is tested in the following regression model:

Regression model: Ordinary least squares.

Dependent variable: Investor Value.

Independent variables: Treatment (Testosterone = 1, Placebo = 0), Basal Testosterone.

Hypothesis: Negative coefficient of Basal Testosterone.

Exploratory Hypothesis 3: The association between basal testosterone and Investor Values will be stronger for participants with lower basal cortisol. This is tested in the following regression model:

Regression model: Ordinary least squares.

Dependent variable: Investor Value.

Independent variables: Treatment (Testosterone = 1, Placebo = 0), Basal Testosterone, Basal Cortisol, Basal Testosterone*Basal Cortisol.

Hypothesis: Positive coefficient of interaction Basal Testosterone*Basal Cortisol.

Exploratory Hypothesis 4: Participants with higher basal testosterone will exhibit higher Trustee Values. This is tested in the following regression model:

Regression model: Ordinary least squares.

Dependent variable: Trustee Value.

Independent variables: Treatment (Testosterone = 1, Placebo = 0), Basal Testosterone.

Hypothesis: Positive coefficient of Basal Testosterone.

Exploratory Hypothesis 5: The association between basal testosterone and Trustee Values will be stronger for participants with lower basal cortisol. This is tested in the following regression model:

Regression model: Ordinary least squares.

Dependent variable: Trustee Value.

Independent variables: Treatment (Testosterone = 1, Placebo = 0), Basal Testosterone, Basal Cortisol, Basal Testosterone*Basal Cortisol.

Hypothesis: Negative coefficient of interaction Basal Testosterone*Basal Cortisol.

4. The dictator game

4.1 Procedure and outcome measures

Procedure. Participants started by reading the instructions for the dictator game, which appeared as follows:

"You will play one round of this game, in which you will be paired with another participant from this study. You will not find out who the other participant is, nor will they find out who you are. In this round, you will receive $10. Your job is to decide how to divide the $10 between yourself and the other participant. You can choose to do one of the following:

a. Keep $10 and give the other participant $0
b. Keep $9 and give the other participant $1
c. Keep $8 and give the other participant $2

d. Keep $7 and give the other participant $3
e. Keep $6 and give the other participant $4
f. Keep $5 and give the other participant $5
g. Keep $4 and give the other participant $6
h. Keep $3 and give the other participant $7
i. Keep $2 and give the other participant $8
j. Keep $1 and give the other participant $9
k. Keep $0 and give the other participant $10

You will receive the amount of money that you decide to keep and the other participant will receive the amount of money that you decide to give to him."

After reading the instructions, participants completed a 2-item task comprehension questionnaire to verify that they fully understood all task dynamics. Upon completion of the comprehension questionnaire, participants immediately began the dictator game and responded to the scenario described in the instructions.

Payment. Participants played a total of thirteen rounds of the dictator game (one round) and charity game (twelve rounds); see more about the charity game below. One of these thirteen rounds was randomly selected for payment. If the single round of the dictator game was selected for a given participant, this participant was randomly paired with another participant. He received the amount of money that he decided to keep. The other participant received the amount of money that he decided to give to him. Participants did not receive immediate feedback about their payment. They were paid approximately one month after they completed the study.

Data Transformations. A Dictator Value was calculated for each participant in accordance with the amount of money that they chose to give to the other participant (e.g. choice a = 0).

4.2 Preregistered hypothesis tests and exploratory analyses

Hypothesis 1: Participants' average Dictator Value will be lower after testosterone administration than after placebo administration. We will test this hypothesis by conducting a two-sample t-test comparing the average Dictator Value in the testosterone group to the average Dictator Value in the placebo group. Our threshold for statistical significance will be alpha = 0.005, whereas our threshold for suggestive evidence will be alpha = 0.05.

Robustness Check: Participant's average Dictator Value will be lower after testosterone administration than after placebo administration when controlling for right-hand 2D:4D, pleasant-

unpleasant mood, arousal-calm mood, time of day, and site. This is tested in the following regression model:

Regression model: Ordered logistic regression.

Dependent variable: Dictator Value.

Independent variables: Treatment (Testosterone = 1, Placebo = 0), Right-Hand 2D:4D, Pleasant-Unpleasant mood, Arousal-Calm Mood, Time of Day, Site.

Hypothesis: Negative coefficient of Treatment.

Exploratory Hypothesis 1: The association between treatment and Dictator Values will be stronger for participants with lower basal cortisol. This is tested in the following regression model:

Regression model: Ordinary least squares.

Dependent variable: Dictator Value.

Independent variables: Treatment (Testosterone = 1, Placebo = 0), Basal Cortisol, Treatment*Basal Cortisol.

Hypothesis: Positive coefficient of interaction Treatment*Basal Cortisol.

Exploratory Hypothesis 2: Dictator Values will be lower for participants with higher basal testosterone. This is tested in the following regression model:

Regression model: Ordered logistic regression.

Dependent variable: Dictator Value.

Independent variables: Treatment (Testosterone = 1, Placebo = 0), Basal Testosterone.

Hypothesis: Negative coefficient of Basal Testosterone.

Exploratory Hypothesis 3: The association between basal testosterone and Dictator Values will be stronger for participants with lower basal cortisol. This is tested in the following regression model:

Regression model: Ordinary least squares.

Dependent variable: Dictator Value.

Independent variables: Treatment (Testosterone = 1, Placebo = 0), Basal Testosterone, Basal Cortisol, Basal Testosterone*Basal Cortisol.

Hypothesis: Positive coefficient of interaction Basal Testosterone*Basal Cortisol.

5. The charity game

5.1 Procedure and outcome measures

Procedure. In each round of this game, participants divided an endowment of \$6, \$8, or \$10 between themselves and North Bay Food Bank (a charity that works within the community to gather and distribute food to those in need), in increments of \$1. A multiplier was assigned for each round, such that North Bay Food Bank received \$0.75, \$1.00, \$1.25, or \$1.75 for every dollar donated. Participants played a total of twelve rounds of this game (3 endowments x 4 multipliers). Rounds were presented in a random order.

Payment. Participants played a total of thirteen rounds of the charity game (twelve rounds) and dictator game (one round). One of these thirteen rounds was randomly selected for payment. If a round of the charity game was selected for a given participant, this participant received the amount of money that he decided to keep in this round. North Bay Food Bank received the amount of money that he decided to donate in this round, multiplied by the corresponding multiplier. Participants did not receive immediate feedback about their payment. They were paid approximately one month after they completed the study.

Data Transformations.

1. Price of Donating: This was calculated as the reciprocal of the multiplier for each round.
2. Percentage Donated: This was calculated by taking the amount of money that a participant decided to donate in a given round, dividing it by the endowment for that round, and then multiplying it by one hundred.

5.2 Pre-registered hypothesis tests and exploratory analyses

All the below tests were pre-registered as the following linear mixed-effect models estimated in R using the “lmer” function from lme4. However, for exploratory analyses 1–3 and the robustness tests of these analyses the models did not converge and we therefore replaced these models with

ordinary least squares regressions with clustering the standard errors on the participant level (due to the multiple 12 observations per participant). For hypothesis one and the robustness test of hypothesis one, the linear mixed-effect models converged; for these models, we also checked that using ordinary least squares regressions with clustering on the standard errors on the participant level produced similar results (and the results are almost identical; for the linear mixed-effect model (ordinary least squares with clustering on participant) in hypothesis one the coefficient, standard error, t-values and p-values are -1.908 (-1.908), 2.560 (2.559), -0.745 (-0.745) and 0.456 (0.456); for the linear mixed-effect model (ordinary least squares with clustering on the participant) in the robustness test of hypothesis one the coefficient, standard error, t-values and p-values are -0.410 (-0.410), 2.742 (2.742), -0.149 (-0.149) and 0.881 (0.881)).

Hypothesis 1: Participants will exhibit greater price inflexibility after testosterone administration than after placebo administration (i.e. they will be more likely to donate the same amount of money, irrespective of price). This is tested in the following regression model:

Regression model: Linear mixed-effect model with 12 observations per participant (Treatment, Treatment*Price of Donating, and Endowment treated as fixed effects; Price of Donating and its intercept treated as random effects for each participant).

Dependent variable: Percentage Donated.

Independent variables: Treatment (Testosterone = 1, Placebo = 0), Price of Donating, Treatment*Price of Donating, Endowment.

Hypothesis: Positive coefficient of interaction Treatment*Price of Donating.

Exploratory Hypothesis 1: Testosterone's effects on price inflexibility will be stronger in men with higher trait impulsivity. This is tested in the following regression model:

Regression model: Ordinary least squares regression with 12 observations per participant and clustering the standard errors on the participant level.

Dependent variable: Percentage Donated.

Independent variables: Treatment (Testosterone = 1, Placebo = 0), Price of Donating, Trait Impulsivity, Treatment*Price of Donating, Treatment*Trait Impulsivity, Price of Donating*Trait Impulsivity, Treatment*Price of Donating*Trait Impulsivity, Endowment.

Hypothesis: Positive coefficient of triple interaction Treatment*Price of Donating*Trait Impulsivity.

Exploratory Hypothesis 2: Testosterone's effects on price inflexibility will be stronger in men who believe they received placebo than in men who believe they received testosterone. This is tested in the following regression model:

Regression model: Ordinary least squares regression with 12 observations per participant and clustering the standard errors on the participant level.

Dependent variable: Percentage Donated.

Independent variables: Treatment (Testosterone = 1, Placebo = 0), Price of Donating, Treatment Belief, Treatment*Price of Donating, Treatment*Treatment Belief, Price of Donating*Treatment Belief, Treatment*Price of Donating*Treatment Belief, Endowment.

Hypothesis: Positive coefficient of triple interaction Treatment*Price of Donating*Treatment Belief.

Exploratory Hypothesis 3: Testosterone treatment will affect percentage donated. This is tested in the following regression model:

Regression model: Ordinary least squares regression with 12 observations per participant and clustering the standard errors on the participant level.

Dependent variable: Percentage Donated.

Independent variables: Treatment (Testosterone = 1, Placebo = 0), Price of Donating, Endowment.

Hypothesis: No directional hypothesis.

Robustness check 1: Participants will exhibit greater price inflexibility after testosterone administration than after placebo administration (i.e. they will be more likely to donate the same amount of money, irrespective of price) when controlling for right-hand 2D:4D, pleasant-

unpleasant mood, arousal-calm mood, time of day, and site. This is tested in the following regression model:

Regression model: Linear mixed-effect model with 12 observations per participant (Treatment, Treatment*Price of Donating, Endowment and the added control variables treated as fixed effects; Price of Donating and its intercept treated as random effects for each participant).

Dependent variable: Percentage Donated.

Independent variables: Treatment (Testosterone = 1, Placebo = 0), Price of Donating, Treatment*Price of Donating, Endowment, Right-Hand 2D:4D, Pleasant-Unpleasant mood, Arousal-Calm Mood, Time of Day, Site.

Hypothesis: Positive coefficient of interaction Treatment*Price of Donating.

Robustness check 2: Testosterone's effects on price inflexibility will be stronger in men with higher trait impulsivity when controlling for right-hand 2D:4D, pleasant-unpleasant mood, arousal-calm mood, time of day, and site. This is tested in the following regression model:

Regression model: Ordinary least squares regression with 12 observations per participant and clustering the standard errors on the participant level.

Dependent variable: Percentage Donated.

Independent variables: Treatment (Testosterone = 1, Placebo = 0), Price of Donating, Trait Impulsivity, Treatment*Price of Donating, Treatment*Trait Impulsivity, Price of Donating*Trait Impulsivity, Treatment*Price of Donating*Trait Impulsivity, Endowment, Right-Hand 2D:4D, Pleasant-Unpleasant mood, Arousal-Calm Mood, Time of Day, Site.

Robustness check 3: Testosterone's effects on price inflexibility will be stronger in men who believe they received placebo than in men who believe they received testosterone when controlling for right-hand 2D:4D, pleasant-unpleasant mood, arousal-calm mood, time of day, and site. This is tested in the following regression model:

Regression model: Ordinary least squares regression with 12 observations per participant and clustering the standard errors on the participant level.

Dependent variable: Percentage Donated.

Independent variables: Treatment (Testosterone = 1, Placebo = 0), Price of Donating, Treatment Belief, Treatment*Price of Donating, Treatment*Treatment Belief, Price of Donating*Treatment Belief, Treatment*Price of Donating*Treatment Belief, Endowment, Right-Hand 2D:4D, Pleasant-Unpleasant mood, Arousal-Calm Mood, Time of Day, Site.

Robustness check 4: Testosterone treatment will affect percentage donated when controlling for right-hand 2D:4D, pleasant-unpleasant mood, arousal-calm mood, time of day, and site. This is tested in the following regression model:

Regression model: Ordinary least squares regression with 12 observations per participant and clustering the standard errors on the participant level.

Dependent variable: Percentage Donated.

Independent variables: Treatment (Testosterone = 1, Placebo = 0), Price of Donating, Endowment, Right-Hand 2D:4D, Pleasant-Unpleasant mood, Arousal-Calm Mood, Time of Day, Site.

Hypothesis: No directional hypothesis for coefficient of Treatment.

6. Competition task

6.1 Procedure and outcome measures

Procedure (General). This task consisted of three parts. In each part, participants were given two minutes to solve as many math problems as possible. Each problem entailed adding together four two-digit numbers (e.g. 63+19+12+44=...). 35 problems were presented in each part. Participants were provided with a pen and paper. Participants were not required to solve these problems in their order of appearance. Each part employed a different payment scheme. One part was randomly selected for payment.

Procedure (Part One). Participants earned $0.50 for each problem that they solved correctly.

Procedure (Part Two). Each participant was paired with an anonymous competitor. If they solved more problems correctly than their anonymous competitor, they earned $0.75 for each problem

that they solved correctly. If they solved fewer problems correctly than their anonymous competitor, they earned $0.00. If there was a tie, they earned $0.50 for each problem that they solved correctly.

Procedure (Part Three). In this part, participants choose between two payment methods.

1. Payment Method One: Participants will earn $0.50 for each problem that they solve correctly.
2. Payment Method Two: Payment will depend on whether participants solve more or fewer problems correctly than their anonymous competitor solved correctly in part two. If they solve more problems correctly, they will earn $0.75 for each problem that they solve correctly. If they solve fewer problems correctly, they will earn $0.00. If there is a tie, they will earn $0.50 for each problem that they solve correctly.

Data Transformations. The number of problems that each participant solved correctly in part one was treated as their Part One Score. The number of problems that each participant solved correctly in part two was treated as their Part Two Score. We calculated a Performance Change Score for each participant by deducting their Part One Score from their Part Two Score. A participant's Willingness to Compete was equal to 0, if they choose payment method one in part three. A participant's Willingness to Compete was equal to 1, if they choose payment method two in part three.

6.2 Pre-registered hypothesis tests and exploratory analyses

Hypothesis 1: Willingness to Compete will be greater after testosterone administration than after placebo administration. We will test this hypothesis by conducting a two-sample z-test of proportions comparing the proportion of participants in the testosterone group that choose payment method two in part three to the proportion of participants in the placebo group that choose payment method two in part three. Our hypothesis implies that the proportion of participants in the testosterone group that choose payment method two in part three will be greater than the proportion of the participants in the placebo group that choose payment method two in part three. Our threshold for statistical significance will be alpha = 0.005, whereas our threshold for suggestive evidence will be alpha = 0.05.

Robustness Check: We predict that Willingness to Compete will be greater after testosterone administration than after placebo administration when controlling for risk aversion, math ability, right-hand 2D:4D, pleasant-unpleasant mood, arousal-calm mood, time of day, and site. This is tested in the following regression model:

Regression model: Logistic regression.

Dependent variable: Willingness to Compete.

Independent variables: Treatment (Testosterone = 1, Placebo = 0), Risk Aversion, Math Ability, Right-Hand 2D:4D, Pleasant-Unpleasant mood, Arousal-Calm Mood, Time of Day, Site.

Hypothesis: Positive coefficient of Treatment.

Exploratory Hypothesis 1: Performance Change Scores will be higher after testosterone administration than after placebo administration. We will test this hypothesis by conducting a two-sample t-test comparing the average Performance Change Score of participants in the testosterone group to the average Performance Change Score of participants in the placebo group. Our hypothesis implies that the average Performance Change Score of participants in the testosterone group will be greater than the average Performance Change Score of participants in the placebo group. Our threshold for statistical significance will be alpha = 0.005, whereas our threshold for suggestive evidence will be alpha = 0.05.

Exploratory Hypothesis 2: The association between treatment and Willingness to Compete will be stronger for participants with higher trait dominance. This is tested in the following regression model:

Regression model: Ordinary least squares.

Dependent variable: Willingness to Compete.

Independent variables: Treatment (Testosterone = 1, Placebo = 0), Trait Dominance, Treatment*Trait Dominance.

Hypothesis: Positive coefficient of interactionTreatment*Trait Dominance.

Exploratory Hypothesis 3: Participants with higher basal testosterone will exhibit greater Willingness to Compete. This is tested in the following regression model:

Regression model: Logistic regression.

Dependent variable: Willingness to Compete.

Independent variables: Treatment (Testosterone = 1, Placebo = 0), Basal Testosterone.

Hypothesis: Positive coefficient of Basal Testosterone.

Exploratory Hypothesis 4: Participants with higher basal cortisol will exhibit lesser Willingness to Compete. This is tested in the following regression model:

Regression model: Logistic regression.

Dependent variable: Willingness to Compete.

Independent variables: Treatment (Testosterone = 1, Placebo = 0), Basal Cortisol.

Hypothesis: Negative coefficient of Basal Cortisol.

Exploratory Hypothesis 5: The association between basal testosterone and Willingness To Compete will be stronger for participants with lower basal cortisol. This is tested in the following regression model:

Regression model: Ordinary least squares.

Dependent variable: Willingness to Compete.

Independent variables: Treatment (Testosterone = 1, Placebo = 0), Basal Testosterone, Basal Cortisol, Basal Testosterone*Basal Cortisol.

Hypothesis: Negative coefficient of Basal Testosterone*Basal Cortisol.

Exploratory Hypothesis 6: The association between treatment and Willingness to Compete will be stronger for participants with lower basal cortisol. This is tested in the following regression model:

Regression model: Ordinary least squares.

Dependent variable: Willingness to Compete.

Independent variables: Treatment (Testosterone = 1, Placebo = 0), Basal Cortisol, Treatment*Basal Cortisol.

Hypothesis: Negative coefficient of interaction Treatment*Basal Cortisol.

Exploratory Hypothesis 7: Participants with higher basal testosterone will exhibit greater Performance Change. This is tested in the following regression model:

Regression model: Ordinary least squares.

Dependent variable: Performance Change Score.

Independent variables: Treatment (Testosterone = 1, Placebo = 0), Basal Testosterone.

Hypothesis: Positive coefficient of Basal Testosterone.

Exploratory Hypothesis 8: Participants with higher basal cortisol will exhibit lesser Performance Change. This is tested in the following regression model:

Regression model: Ordinary least squares.

Dependent variable: Performance Change Score.

Independent variables: Treatment (Testosterone = 1, Placebo = 0), Basal Cortisol.

Hypothesis: Negative coefficient of Basal Cortisol.

Exploratory Hypothesis 9: The association between basal testosterone and Performance Change will be stronger for participants with lower basal cortisol. This is tested in the following regression model:

Regression model: Ordinary least squares.

Dependent variable: Performance Change Score.

Independent variables: Treatment (Testosterone = 1, Placebo = 0), Basal Testosterone, Basal Cortisol, Basal Testosterone*Basal Cortisol.

Hypothesis: Negative coefficient of interaction Basal Testosterone*Basal Cortisol.

Exploratory Hypothesis 10: The association between treatment and Performance Change will be stronger for participants with lower basal cortisol. This is tested in the following regression model:

Regression model: Ordinary least squares.

Dependent variable: Performance Change Score.

Independent variables: Treatment (Testosterone = 1, Placebo = 0), Basal Cortisol, Treatment*Basal Cortisol.

Hypothesis: Negative coefficient of interaction Treatment*Basal Cortisol.

7. Non-preregistered exploratory analysis

As part of the risk task risk-taking was also assessed on a 0–10 scale based on the following question from Dohmen et al. (7):

"Please indicate your willingness to take financial risks, in general, by choosing a number from 0 to 10. 0 represents completely unwilling and 10 represents completely willing."

Based on this question carry out the below exploratory analysis:

Non-preregistered exploratory analysis: Participants will exhibit more risk taking after testosterone administration than after placebo administration. An independent-samples t-test will be used to test this hypothesis. Our threshold for statistical significance will be alpha = 0.005, whereas our threshold for suggestive evidence will be alpha = 0.05.

8. Non-preregistered robustness tests and exploratory analyses.

Based on suggestions from reviewers, we also conduct four sets of robustness tests and exploratory analyses that were not pre-registered. In a first set of robustness tests we add controls (fixed effects) for year and month of the data collection in all our regression analyses described above. In the regressions not already including a control for time of day, we also add time of day

as a control so that we control for time of day, month and year in all the regression analyses. These results are reported in Tables S13-S20. In an additional set of robustness tests, we exclude outliers in the basal salivary testosterone variable excluding participants with a basal salivary testosterone value (pg/ml) >150 (83 participants). In this set of robustness tests, we re-estimate all the exploratory analyses testing for an effect of basal salivary testosterone (including the tests of interaction effects between basal salivary testosterone and other variables). The results are reported in Tables S21-S25. In the third set, we test for non-linearities in the exploratory analyses testing for an association between basal salivary testosterone and the outcome measures by adding the square of basal salivary testosterone to the regressions. We carry out these analyses both with and without the outliers in basal salivary testosterone and these results are reported in SI Appendix Tables S26-S27. In the final set, we test for heterogenous treatment effects by adding an interaction between treatment and basal salivary testosterone to the exploratory analyses testing for an association between basal salivary testosterone and the outcome measures. We carry out these analyses both with and without the outliers in basal salivary testosterone and these results are reported in SI Appendix Tables S28-S29. All the regressions involving interactions are estimated as ordinary least squares regressions.

9. Definition of independent variables in the above analyses

For some of these variables there were some changes in the definitions due to ambiguity in the preregistration or due to these variables not being collected and coded in the raw data exactly as pre-registered. Any such changes are mentioned explicitly below in defining the variables.

Treatment. Dummy variable for the treatment group (Testosterone = 1, Placebo = 0).

Price of Donating, The reciprocal of the multiplier for each round in the charity game.

Endowment: The endowment in each round of the charity game.

Investment Amount: The investment amount in part two of the trust game that the trustees base their back-transfer decision on. It is varied in the five rounds as trustee in the trust game (the investment amount is 2 for round one in part two, 6 for rounds two and three in part two, and 10 for rounds four and five in part two).

Math Ability. Assessed as part of a task gauging participants' willingness to compete (part one of this task). Participants were given two minutes to solve as many math equations (out of thirty-

five) as possible (e.g. 63+19+12+44=...), with the potential to earn $0.50 for each correct answer, using a provided pen and paper. Math ability was calculated as the total number of correct answers provided by each participant.

Right-Hand 2D:4D. Calculated as the ratio of each participant's right-hand index finger length to right-hand ring finger length. Each participant's right-hand was scanned via the use of a printer in the soak period after the treatment administration, but before the primary experimental tasks. Finger length in scans was measured by two coders using a digital ruler. We used the average ratio of the two measurements by the two coders as our measure of right-hand 2D:4D; but the preregistration did not specify how the measurements of the two raters would be combined.

Pleasant-Unpleasant Mood. Assessed via the Brief Mood Introspection Scale measured as part of the primary experimental tasks after the treatment administration.

Arousal-Calm Mood. Assessed via the Brief Mood Introspection Scale measured as part of the primary experimental tasks after the treatment administration.

Treatment Belief. Assessed by asking participants "If you had to guess whether you received testosterone or placebo (a substance with no effect), what would you guess? a. I received testosterone b. I received placebo (a substance with no effect)". Coded as choice a (testosterone) = 0, and choice b (placebo) = 1. This question was asked at the end of the experiment after the experimental tasks.

Self-Construal. Measured via the use of the Self-Construal Scale, by subtracting interdependence from independence. Measured at baseline before the treatment administration.

Trait Dominance. Measured via the Self-Report Dominance and Prestige Scale measured at baseline before the treatment administration.

Trait Impulsivity. Trait impulsivity was calculated as follows. First, each participant's score for the Barratt Impulsivity Scale was normalized (z-scored) within the study sample (the Barrat impulsivity scale was measured at baseline before the treatment administration). Then, each participant's score for the Brief Self-Control Scale was reversed and normalized (z-scored) within the study sample (the Brief Self-Control Scale was measured at baseline before the treatment administration). To create our trait impulsivity index, we added each participant's normalized and reversed score for the Brief-Self Control Scale to their normalized score for the Barratt Impulsivity Scale.

Waking Time (used in estimating basal testosterone and basal cortisol below). Assessed by asking participants "When did you wake up today (e.g. if you woke up at 7:30 A.M.,

please write 7:30)?". We coded this variable based on the following eight categories and seven dummy variables: Baseline: <6:00; Dummy 1: 6:00 to 6:59; Dummy 2: 7:00 to 7:59; Dummy 3: 8:00 to 8:59; Dummy 4: 9:00 to 9:59; Dummy 5: 10:00 to 10:59; Dummy 6: 11:00 to 11:59; Dummy 7: 12:00 or later. The coding of the variable was not specified in the preregistration.

Time of Day. It was pre-registered that sessions would take place at 9:30 AM, 12:00 PM, 2:30 PM, and 5:30 PM and that we to control for time of day fixed effects would create a dummy variable for each of these session times (treating 9:30 AM as our baseline, and three dummy variables for 12:00 PM, 2:30 PM, and 5:30 PM). However, the sessions (data collection) were started at different times during the day and the exact starting time was not recorded in the data collection, making it hard to assign observations to these four exact times. But the time of the first (pre-treatment) saliva sample was recorded in the data collection and based on this we used the four following categories, with three dummy variables: Baseline: 08:00 to 10:44; Dummy 1: 10:45 to 13:14; Dummy 2: 13:15 to 15:59; Dummy 3: 16:00 to 20:00.

Site. Sessions took place at Nipissing University's Social Neuroendocrinology Laboratory in North Bay, Ontario, Georgian College in Barrie, Ontario, and Medical Offices in Sudbury, Ontario. To control for site fixed effects, we created a binary variable corresponding to if the data was collected at Nipissing University (coded as 1) or on another study location coded as (0). Data were only planned to be collected at two sites and the pre-registration defined "Site" as a dummy variable defined as (0 = Nipissing, 1 = Northeastern); and as a third site was added during the study it is coded as "another study location" in the "Site" variable (to define this variable as closely as possible to the definition in the preregistration).

Basal Testosterone. Basal testosterone (pg/ml) was determined via the analysis of pre-treatment saliva samples using commercially-available enzyme immunoassay kits from DRG International. The standard curve of this assay kit ranges from 0 to 1,000 pg/ml. Samples with concentrations above 1,000 pg/ml were flagged as potentially contaminated by residual gel within the testing area. We implemented a procedure aimed at minimizing the possibility of sample contamination which consisted of using disposable keyboard and mouse covers, as well as the use of 70% ethanol to clean hard surfaces such as doorknobs and tables. This cleanup procedure was largely effective as only 0.5% (5 observations) of baseline samples were outside the range of the assay kit. We estimated an ordinary least squares regression model with the log of basal testosterone level as the dependent variable and waking time and time of day as independent variables. Basal testosterone was defined as the residuals of this regression. The preregistration incorrectly stated that the regression would be run with basal testosterone level as the dependent variable and that the variable would be defined as the log of the residuals of this regression; but as it is impossible to log negative residuals the regression was run with the log of basal

testosterone level as the dependent variable. In SI Appendix, Table S1 with descriptives at baseline we report this variable in pg/ml before pre-processing.

Basal Cortisol: Basal cortisol (ng/ml) was determined via the analysis of pre-treatment saliva samples (ELISA). Basal cortisol levels were pre-processed as follows. We estimated an ordinary least squares regression model with the log of basal cortisol level as the dependent variable and waking time and time of day as independent variables. Basal cortisol was defined as the residuals of this regression. The preregistration incorrectly stated that the regression would be run with basal cortisol level as the dependent variable and that the variable would be defined as the log of the residuals of this regression; but as it is impossible to log negative residuals the regression was run with the log of basal cortisol level as the dependent variable. In SI Appendix, Table S1 with descriptives at baseline we report this variable in ng/ml before pre-processing.

Year: Variable for the year of the data collection with 2018 as the baseline category and five dummy variables for 2019, 2020, 2021, 2022, and 2023. This variable was not part of any pre-registered analysis, but included in non-preregistered robustness tests.

Month: Variable for the month of the data collection with January as the baseline category and 11 dummy variables for the remaining 11 months. This variable was not part of any pre-registered analysis, but included in non-preregistered robustness tests.

SI References

1. Ai C, Norton EC. Interaction terms in logit and probit models. Econ Lett. 2003 Jul 1;80(1):123–9.

2. Chapman J, Snowberg E, Wang S, Camerer C. Loss Attitudes in the U.S. Population: Evidence from Dynamically Optimized Sequential Experimentation (DOSE). 2018.

3. Chumbley JR, Krajbich I, Engelmann JB, Russell E, Van Uum S, Koren G, et al. Endogenous Cortisol Predicts Decreased Loss Aversion in Young Men. Psychol Sci. 2014 Nov 1;25(11):2102–5.

4. Krajbich I, Camerer C, Rangel A. Exploring the scope of neurometrically informed mechanism design. Games Econ Behav. 2017 Jan 1;101:49–62.

5. Chapman J, Snowberg E, Wang SW, Camerer C. Looming Large or Seeming Small? Attitudes Towards Losses in a Representative Sample. Rev Econ Stud. 2024 Sep 16;rdae093.

6. Tversky A, Kahneman D. Advances in prospect theory: Cumulative representation of uncertainty. J Risk Uncertain. 1992 Oct 1;5(4):297–323.

7. Dohmen T, Falk A, Huffman D, Sunde U, Schupp J, Wagner GG. Individual Risk Attitudes: Measurement, Determinants, and Behavioral Consequences. J Eur Econ Assoc. 2011 Jun 1;9(3):522–50.

8. Eisenegger C, Naef M, Snozzi R, Heinrichs M, Fehr E. Prejudice and truth about the effect of testosterone on human bargaining behaviour. Nature. 2010 Jan 1;463(7279):356–9.

SI Tables

Table S1. Key results in previous related studies on testosterone and economic preferences, with the relevant outcome variable in our study listed within parentheses (we only include studies testing effects on the same outcome measures as in our study).

Correlational studies		
**Study**	**N[#]**	**Finding[&]**
Burnham (2007)	26 men	Ultimatum game rejections (Responder Value) positively correlated with testosterone in men.
Apicella et al. (2008)	88 men	Testosterone positively correlated with risk-taking (Risk Aversion*) in men.
Sapienza, Zingales & Maestripieri (2009)	320 men, 140 women	Testosterone positively correlated with risk-taking (Risk Aversion[#]) in women but not in men.
Stanton et al. (2011)	142 men, 156 women	A U-shaped relationship between testosterone and risk taking (Risk Aversion*) in both men and women.
Eisenegger et al. (2017)	172 men	Testosterone positively correlated with willingness to compete (Willingness to Compete) in men.
Stanton et al.(2021)	94 men, 119 women	No association between testosterone and risk taking (Risk Aversion*) in men or women.
Schipper (2023)	115 men, 93 women	Testosterone positively correlated with risk-taking (Risk Aversion*) in men but not in women.
Novakova et al. (2024)	74 men, 84 women	Testosterone negatively correlated with dictator game giving (Dictator Value) in women and positively correlated in men.
Massacsesi et al. (2024	499 men, 503 women	No association between testosterone and dictator game (Dictator Value), trust game (Investor Value and Trustee Value), or ultimatum game (Proposer Value and Responder Value).
**Studies exogenously manipulating testosterone**		
**Study**	**N**	**Finding**
Zak et al. (2009)	25 men	Testosterone decreased offers (Proposer Value) in the ultimatum game in men, no effect on dictator game giving (Dictator Value).
Zethraeus et al. (2009)	134 women	No effect of testosterone on risk-taking (Risk Aversion*), ultimatum game (Proposer Value and Responder Value), dictator game (Dictator Value), or trust game (Investor Value and Trustee Value) in women.
Eisenegger et al. (2010)	121[†] women	Testosterone increased offers (Proposer Value) in the ultimatum game in women, no effect on ultimatum game rejections (Responder Value).
Boksem et al. (2013)	54 women	Testosterone decreased trust (Investor Value) and increased trustworthiness (Trustee Value) in women in the trust game, no effect on risk taking (Risk Aversion*).

Buskens et al. (2016)	82 women	No effect of testosterone on trust (Investor Value) in women in the trust game.
Dreher et al. (2016)	40 men	No effect of testosterone on ultimatum game rejections (Responder Value) in men.
Kopsida et al. (2016)	40 men, 28 women	No effect of testosterone on ultimatum game rejections (Responder Value) in men and women.
Cueva et al. (2017)	41 men	No effect of testosterone on ultimatum game rejections (Responder Value) in men.
Geniole et al. (2019)	118 men	No effect of testosterone on offers (Proposer Value) in the ultimatum game in men.
Stanton et al. 2021	143 men (in two studies)	No effect of testosterone on risk-taking (Risk Aversion*) or loss aversion (Loss Aversion) in men.
Nadler et al. (2024)	333 men (in two studies)	No effect of testosterone on risk-taking (Risk Aversion*) or willingness to compete (Willingness to Compete) in men.

[#]The effective sample size is in some analyses smaller than the one reported in this column. In addition, some studies have between-participant and some within-participant designs.
[&]Many of the studies report significant interaction effects or other significant results but here we only report the main effects included as primary hypotheses in our study.
[*]Note that a higher risk aversion parameter implies less risk aversion.
[†]The sample for the proposer analysis is N=60.

.

Table S2. Descriptives measured at baseline in the placebo group and the testosterone group.

Variable	Placebo group			Testosterone group		
	Mean	Standard Deviation	n	Mean	Standard Deviation	n
Basal testosterone (pg/ml)#	77.64	146.4	496	122.92	479.8	498
Log basal testosterone (pg/ml)#	4.091	0.637	496	4.306	0.723	498
Preprocessed basal testosterone#	-0.009	0.747	488	0.007	0.605	492
Basal cortisol (ng/ml)#	1.874	2.476	483	1.674	1.629	489
Log basal cortisol (ng/ml)#	0.194	0.998	483	0.130	0.986	489
Preprocessed basal cortisol#	0.013	0.884	475	0.029	0.966	483
Age (years)	23.67	5.709	499	24.37	6.254	499
Right-hand 2D:4D	0.947	0.030	458	0.945	0.029	450
Self-construal	0.172	1.003	499	0.361	0.957	500
Trait impulsivity	0.019	1.81	497	-0.023	1.874	500
Trait dominance	3.345	1.011	499	3.418	1.047	500
Waking time <6:00	0.052	0.223	496	0.054	0.227	498
Waking time 6:00-6:59	0.155	0.363	496	0.159	0.366	498
Waking time 7:00-7:59	0.288	0.453	496	0.269	0.444	498
Waking time 8:00-8:59	0.258	0.438	496	0.249	0.433	498
Waking time 9:00-9:59	0.129	0.336	496	0.127	0.333	498
Waking time 10:00-10:59	0.056	0.231	496	0.090	0.287	498
Waking time 11:00-11:59	0.032	0.177	496	0.038	0.192	498
Waking time ≥12	0.028	0.166	496	0.014	0.118	498
Time of day 08:00-10:44	0.266	0.442	496	0.234	0.424	496
Time of day 10:45-13:14	0.323	0.468	496	0.290	0.454	496
Time of day 13:15-15:59	0.282	0.451	496	0.317	0.466	496
Time of day 16:00-20:00	0.129	0.336	496	0.151	0.359	496
Site (Nipissing=1)	0.729	0.445	499	0.740	0.439	500

Note that the baseline testosterone and basal cortisol variables used in the analyses are preprocessed to control for time of day and waking time; but in this Table we show both the mean values before preprocessing (with and without taking logs of the variable) and the preprocessed values; see SI Appendix, section 9.

Table S3. Descriptives for testosterone, outcome variables and independent variables in the placebo group and the testosterone group measured after the administration of testosterone or placebo.

Variable	Placebo group			Testosterone group		
	**Mean**	**Standard Deviation**	**n**	**Mean**	**Standard Deviation**	**n**
Testosterone (post intervention) (pg/ml)	97.93	453.1	497	4098.11	4303.9	498
Log testosterone (post intervention) (pg/ml)	4.134	0.6484	497	7.235	1.7486	498
Risk Aversion	1.035	0.242	482	1.054	0.235	472
Loss Aversion	2.276	1.016	482	2.308	0.986	472
Proposer Value	3.848	1.130	499	3.903	1.115	500
Responder Value	0.503	0.326	499	0.518	0.328	500
Investor Value	4.766	2.530	499	4.845	2.519	500
Trustee Value	0.316	0.156	499	0.320	0.148	500
Dictator Value	3.459	2.112	499	3.422	2.067	500
Willingness to Compete	0.363	0.481	499	0.382	0.486	500
Percentage Donated	42.72	30.66	5988	41.60	30.20	6000
Performance Change Score	0.854	3.148	499	0.850	2.743	500
Math Ability	6.832	3.402	499	7.076	3.066	500
Pleasant-unpleasant mood	48.13	5.828	491	47.88	6.106	497
Arousal-calm mood	26.86	4.145	491	26.80	4.055	497
Treatment belief (1=placebo)	0.580	0.494	495	0.629	0.484	490

Table S4. Robustness tests of the nine primary hypotheses. Regressions controlling for right-hand 2D:4D, pleasant-unpleasant mood, arousal-calm mood, time of day, and site (and for Willingness to compete controlling also for Risk Aversion and math ability).[#]

Outcome variable	Hypothesized sign	n	Treatment regression coefficient (SE)	Standard error	t/z-value (df)	p-value
Risk Aversion[&]	Positive	846	0.026	0.017	1.598 (837)	0.111
Loss Aversion	Negative	846	0.0088	0.069	0.127 (837)	0.899
Proposer Value	Positive	890	0.104	0.077	1.353 (881)	0.176
Responder Value	Positive	890	0.104	0.122	0.854	0.393
Investor Value	Negative	846	-0.024	0.174	-0.137 (836)	0.891
Trustee Value	Positive	890	0.0056	0.010	0.556 (881)	0.579
Dictator Value	Negative	890	-0.040	0.124	-0.320	0.748
Willingness to Compete	Positive	846	-0.033	0.150	-0.225	0.822
Percentage Donated (Price of Donating* Treatment interaction)	Positive	10680	-0.410	2.742	-0.149 (888)	0.881

[#] Ordinary least squares regression for Proposer Value, Investor Value, Trustee Value, Risk Aversion and Loss Aversion; logistic regression for Willingness to Compete; ordered logistic regression for Dictator Value and Responder Value; a linear mixed-effect model for Percentage Donated (every participant is included with 12 observations in this regression explaining the high n).

[&] Note that a higher risk aversion parameter implies less risk aversion and the hypothesized positive sign thus implies that testosterone is hypothesized to decrease risk aversion.

Table S5. Robustness tests of Proposer Value and Responder Value controlling for "treatment belief" (1=placebo; 0=testosterone) in addition to the control variables included in SI Appendix, Table S3.[#]

	Dependent variable: Proposer Value					
**Independent variable**	**Hypothesized sign**	**n**	**Regression coefficient**	**Standard error**	**t-value (df)**	**p-value**
Treatment	Positive	879	0.100	0.077	1.297 (869)	0.195
Treatment belief (1=placebo)	No hypothesis made in the PAP	879	0.186	0.081	2.308 (869)	0.021
	**Dependent variable: Responder Value**					
**Independent variable**	**Hypothesized sign**	**n**	**Regression coefficient**	**Standard error**	**z-value**	**p-value**
Treatment	Positive	879	0.105	0.123	0.850	0.395
Treatment belief (1=placebo)	No hypothesis made in the PAP	879	0.050	0.128	0.389	0.698

[#] Ordinary least squares regression for Proposer Value and ordered logistic regression for Responder Value. No hypotheses about the sign were made in the pre-analysis plan for "treatment belief"; but Eisenegger et al. (8) reported a statistically significant effect of treatment belief on proposer value with a positive sign (they reported a negative sign but coded it as 1=testosterone, which corresponds to a positive sign with our coding).

Table S6. Exploratory analyses for the risk task (Risk Aversion and Loss Aversion outcome measures). All nine exploratory analysis tests are carried out in separate regressions.[#]

	Dependent variable: Risk Aversion[&]					
**Independent variable**	**Hypothesized sign**	**n**	**Regression coefficient**	**Standard error**	**t-value (df)**	**p-value**
Basal testosterone	Positive	935	-0.0125	0.013	-0.992 (932)	0.321
Basal cortisol	Negative	914	0.0102	0.0080	1.270 (911)	0.204
Basal testosterone* Basal cortisol	Negative	913	0.0091	0.016	0.578 (908)	0.563
Treatment* basal cortisol	Negative	914	-0.009	0.016	-0.547 (910)	0.584
Treatment* Self-construal	Positive	954	0.0094	0.016	0.590 (950)	0.555
	**Dependent variable: Loss Aversion**					
**Independent variable**	**Hypothesized sign**	**n**	**Regression coefficient**	**Standard error**	**t-value (df)**	**p-value**
Basal testosterone	Negative	935	0.024	0.046	0.512 (932)	0.609
Basal cortisol	Positive	914	0.032	0.036	0.893 (911)	0.372
Basal testosterone* Basal cortisol	Positive	913	0.024	0.060	0.399 (908)	0.690
Treatment* Basal cortisol	Positive	914	-0.0065	0.074	-0.089 (910)	0.929

[#] Ordinary least squares regression for all Risk Aversion and Loss Aversion regressions.

[&] Note that a higher risk aversion parameter implies less risk aversion and the hypothesized positive sign thus implies that testosterone is hypothesized to decrease risk aversion.

Table S7. Exploratory analyses for the ultimatum game (Proposer Value and Responder Value outcome measures). All five exploratory analysis tests are carried out in separate regressions.[#]

	**Dependent variable: Proposer Value**					
**Independent variable**	**Hypothesized sign**	**n**	**Regression coefficient**	**Standard error**	**t-value (df)**	**p-value**
Basal testosterone	Positive	979	0.100	0.054	1.850 (976)	0.060
Basal testosterone* Basal cortisol	Negative	956	-0.025	0.067	-0.372 (951)	0.710
	**Dependent variable: Responder Value**					
**Independent variable**	**Hypothesized sign**	**n**	**Regression coefficient**	**Standard error**	**t/z-value (df)**	**p-value**
Basal testosterone	Positive	979	-0.092	0.089	-1.035	0.301
Basal testosterone* Basal cortisol	Negative	956	0.027	0.021	1.288 (951)	0.198
Treatment* Basal cortisol	Negative	957	0.031	0.023	1.336 (953)	0.182

[#] Ordinary least squares regression for all Proposer Value regressions and all Responder Value regressions except the one for "basal testosterone" and Responder Value based on an ordered logistic regression (as that regression does not include any interactions).

Table S8. Exploratory analyses for the trust game (Investor Value and Trustee Value outcome measures). All five exploratory analysis tests are carried out in separate regressions.[#]

	Dependent variable: Investor Value					
**Independent variable**	**Hypothesized sign**	**n**	**Regression coefficient**	**Standard error**	**t-value (df)**	**p-value**
Basal testosterone	Negative	979	0.166	0.123	1.348 (976)	0.178
Basal testosterone* Basal cortisol	Positive	956	-0.030	0.152	-0.201 (951)	0.841
	**Dependent variable: Trustee Value**					
**Independent variable**	**Hypothesized sign**	**n**	**Regression coefficient**	**Standard error**	**t-value (df)**	**p-value**
Basal testosterone	Positive	979	-0.0076	0.0065	-1.162 (976)	0.246
Basal testosterone* Basal cortisol	Negative	956	-0.00017	0.013	-0.013 (951)	0.989
Treatment* Investment amount	Positive	4995	0.00033	0.0020	0.165 (997)	0.869

[#] Ordinary least squares regression for all Investor Value regressions and all Trustee Value regressions except the one for “treatment*investment amount” based on a linear mixed-effect model (as that regression does not include any interactions); in the linear mixed-effect model five observations per participant are included explaining the high n.

Table S9. Exploratory analyses for the dictator game (Dictator Value outcome measure). All three exploratory analysis tests are carried out in separate regressions.#

	**Dependent variable: Dictator Value**					
**Independent variable**	**Hypothesized sign**	**n**	**Regression coefficient**	**Standard error**	**t/z-value (df)**	**p-value**
Basal testosterone	Negative	979	-0.0009	0.088	-0.0102	0.9918
Basal testosterone* Basal cortisol	Positive	956	0.052	0.126	0.408 (951)	0.683
Treatment* Basal cortisol	Positive	957	-0.151	0.149	-1.015 (953)	0.310

Ordinary least squares regression for all Dictator Value regressions except the one for “basal testosterone” based on an ordered logistic regression.

Table S10. Exploratory analyses for the charity game (Percentage Donated outcome measure). All three exploratory analysis tests and the three additional robustness tests of these results are carried out in separate regressions.[#]

	Dependent variable: Percentage Donated					
**Independent variable**	**Hypothesized sign**	**n**	**Regression coefficient**	**Standard error**	**t-value (df)**	**p-value**
Treatment	No hypothesis made in the PAP	11988	-1.123	1.507	-0.745 (997)	0.457
Treatment* Price of donating*Trait impulsivity	positive	11952	-0.897	1.390	-0.646 (364)	0.519
Treatment*Price of donating*Treatment belief	Positive	11820	1.579	5.310	0.297 (817)	0.766
	**Dependent variable: Percentage Donated (robustness test)**					
**Independent variable**	**Hypothesized sign**	**n**	**Regression coefficient**	**Standard error**	**t-value (df)**	**p-value**
Treatment	No hypothesis made in the PAP	10680	-0.110	1.593	-0.069 (880)	0.945
Treatment*Price of donating*Trait impulsivity	Positive	10656	-0.709	1.486	-0.477 (321)	0.633
Treatment*Price of donating*Treatment belief	Positive	10548	1.352	5.639	0.240 (736)	0.811

[#] Ordinary least squares regression with clustering of standard errors on the participant for all Percentage Donated regressions (a linear mixed-effect model regression was preregistered for these regressions, but these regressions failed to converge). Every participant is included with 12 observations in the regression explaining the high n. The analyses in the last three rows are preregistered robustness tests of the three exploratory analyses controlling for: right-hand 2D:4D, pleasant-unpleasant mood, arousal-calm mood, time of day, and site (note that adding these control variables affects the n in the regressions).

Table S11. Exploratory analyses for the competition task (Willingness to Compete and Performance Change Score outcome measures). The ten exploratory analysis tests are carried out in nine separate regressions and one independent samples t-test.#

	Dependent variable: Willingness to Compete					
**Independent variable**	**Hypothesized sign**	**n**	**Regression coefficient**	**Standard error**	**t/z-value (df)**	**p-value**
Basal testosterone	Positive	979	0.190	0.098	1.939	0.052
Basal cortisol	Negative	957	0.084	0.074	1.147	0.251
Basal testosterone* Basal cortisol	Negative	956	0.011	0.030	0.369 (951)	0.712
Treatment* Basal cortisol	Negative	957	-0.045	0.034	-1.324 (953)	0.186
Treatment* Trait dominance	Positive	998	-0.040	0.030	-1.346 (994)	0.179
	**Dependent variable: Performance Change Score**					
**Independent variable**	**Hypothesized sign**	**n**	**Regression coefficient**	**Standard error**	**t/z-value (df)**	**p-value**
Basal testosterone	Positive	979	0.150	0.199	0.758 (976)	0.449
Basal cortisol	Negative	957	0.020	0.120	0.169 (954)	0.866
Basal testosterone* Basal cortisol	Negative	956	0.463	0.231	2.001 (951)	0.046
Treatment* Basal cortisol	Negative	957	-0.218	0.240	-0.908 (953)	0.364
	**Dependent variable: Performance Change Score**					
**Independent variable**	**Hypothesized sign**	**n**	**Treatment effect**	**Standard error**	**t-value (df)**	**p-value**
Treatment	Positive	999	0.0037	0.187	0.020 (997)	0.984

Ordinary least squares regression for all Performance Change Score regressions but the treatment effect on the Performance Change Score on the last row based on a two-sample t-test. The Willingness to Compete regressions without any interactions are estimated with logistic regression and the regressions with interactions estimated with ordinary least squares.

Table S12. Non-preregistered exploratory analysis testing for a treatment effect on an alternative measure of risk taking. An independent samples t-test.

**Outcome variable**	**Hypothesized sign**	**n**	**Treatment effect (SE)**	**Standard error**	**t-value (df)**	**p-value**
Willingness to take financial risks on 0 (completely unwilling) to 10 (completely willing) scale.	Positive	954	-0.091	0.152	-0.599 (952)	0.549

Table S13. Non-preregistered robustness tests of Table S4 results adding controls for year and month of data collection.#

Outcome variable	Hypothesized sign	n	Treatment regression coefficient (SE)	Standard error	t/z-value (df)	p-value
Risk Aversion&	Positive	846	0.023	0.017	1.37 (821)	0.171
Loss Aversion	Negative	846	0.000	0.070	-0.006 (821)	0.996
Proposer Value	Positive	890	0.098	0.078	1.247 (865)	0.213
Responder Value	Positive	879	0.086	0.124	0.697	0.486
Investor Value	Negative	846	-0.054	0.173	-0.312 (820)	0.755
Trustee Value	Positive	890	0.008	0.0104	0.740 (865)	0.459
Dictator Value	Negative	890	-0.027	0.125	-0.218	0.827
Willingness to Compete	Positive	846	-0.079	0.150	-0.525	0.599
Percentage Donated (Price of Donating* Treatment interaction)	Positive	10680	-0.41	2.742	-0.149 (888)	0.881

Ordinary least squares regression for Proposer Value, Investor Value, Trustee Value, Risk Aversion and Loss Aversion; logistic regression for Willingness to Compete; ordered logistic regression for Dictator Value and Responder Value; a linear mixed-effect model for Percentage Donated (every participant is included with 12 observations in this regression explaining the high n).

& Note that a higher risk aversion parameter implies less risk aversion and the hypothesized positive sign thus implies that testosterone is hypothesized to decrease risk aversion.

Table S14. Non-preregistered robustness tests of Table S5 results adding controls for year and month of data collection.#

	Dependent variable: Proposer Value					
**Independent variable**	**Hypothesized sign**	**n**	**Regression coefficient**	**Standard error**	**t-value (df)**	**p-value**
Treatment	Positive	879	0.091	0.079	1.157 (853)	0.247
Treatment belief (1=placebo)	No hypothesis made in the PAP	879	0.185	0.083	2.232 (853)	0.026
	**Dependent variable: Responder Value**					
**Independent variable**	**Hypothesized sign**	**n**	**Regression coefficient**	**Standard error**	**z-value**	**p-value**
Treatment	Positive	879	0.089	0.125	0.711	0.477
Treatment belief (1=placebo)	No hypothesis made in the PAP	879	0.047	0.13	0.362	0.717

Ordinary least squares regression for Proposer Value and ordered logistic regression for Responder Value. No hypotheses about the sign were made in the pre-analysis plan for “treatment belief”; but Eisenegger et al. (8) reported a statistically significant effect of treatment belief on proposer value with a positive sign (they reported a negative sign but coded it as 1=testosterone, which corresponds to a positive sign with our coding).

Table S15. Non-preregistered robustness tests of Table S6 results adding controls for time of day, year and month of data collection.[#]

	**Dependent variable: Risk Aversion[&]**					
**Independent variable**	**Hypothesized sign**	**n**	**Regression coefficient**	**Standard error**	**t-value (df)**	**p-value**
Basal testosterone	Positive	935	-0.017	0.014	-1.175 (913)	0.240
Basal cortisol	Negative	914	0.013	0.008	1.596 (892)	0.111
Basal testosterone* Basal cortisol	Negative	913	0.009	0.016	0.565 (889)	0.572
Treatment* Basal cortisol	Negative	914	-0.010	0.016	-0.59 (891)	0.555
Treatment* Self-construal	Positive	946	0.007	0.016	0.455 (923)	0.65
	**Dependent variable: Loss Aversion**					
**Independent variable**	**Hypothesized sign**	**n**	**Regression coefficient**	**Standard error**	**t-value (df)**	**p-value**
Basal testosterone	Negative	935	0.030	0.050	0.595 (913)	0.552
Basal cortisol	Positive	914	0.028	0.038	0.743 (892)	0.458
Basal testosterone* Basal cortisol	Positive	913	0.050	0.060	0.839 (889)	0.402
Treatment* Basal cortisol	Positive	914	-0.015	0.074	-0.202 (891)	0.840

[#] Ordinary least squares regression for all Risk Aversion and Loss Aversion regressions.

[&] Note that a higher risk aversion parameter implies less risk aversion and the hypothesized positive sign thus implies that testosterone is hypothesized to decrease risk aversion.

Table S16. Non-preregistered robustness tests of Table S7 results adding controls for time of day, year and month of data collection.[#]

	**Dependent variable: Proposer Value**					
**Independent variable**	**Hypothesized sign**	**n**	**Regression coefficient**	**Standard error**	**t-value (df)**	**p-value**
Basal testosterone	Positive	979	0.113	0.057	1.991 (957)	0.047
Basal testosterone* Basal cortisol	Negative	956	0.003	0.070	0.048 (932)	0.962
	**Dependent variable: Responder Value**					
**Independent variable**	**Hypothesized sign**	**n**	**Regression coefficient**	**Standard error**	**t/z-value (df)**	**p-value**
Basal testosterone	Positive	979	-0.119	0.097	-1.232	0.218
Basal testosterone* Basal cortisol	Negative	956	0.030	0.021	1.422 (932)	0.155
Treatment* Basal cortisol	Negative	957	0.030	0.023	1.281 (934)	0.200

[#] Ordinary least squares regression for all Proposer Value regressions and all Responder Value regressions except the one for “basal testosterone” and Responder Value based on an ordered logistic regression (as that regression does not include any interactions).

Table S17. Non-preregistered robustness tests of Table S8 results adding controls for time of day, year and month of data collection.#

	**Dependent variable: Investor Value**					
**Independent variable**	**Hypothesized sign**	**n**	**Regression coefficient**	**Standard error**	**t-value (df)**	**p-value**
Basal testosterone	Negative	979	0.193	0.134	1.438 (957)	0.151
Basal testosterone* Basal cortisol	Positive	944	-0.187	0.169	-1.106 (920)	0.269
	**Dependent variable: Trustee Value**					
**Independent variable**	**Hypothesized sign**	**n**	**Regression coefficient**	**Standard error**	**t-value (df)**	**p-value**
Basal testosterone	Positive	979	-0.001	0.007	-0.200 (957)	0.841
Basal testosterone* Basal cortisol	Negative	956	0.004	0.013	0.273 (932)	0.785
Treatment* Investment amount	Positive	4995	0.000	0.002	0.097 (990)	0.923

Ordinary least squares regression for all Investor Value regressions and all Trustee Value regressions (the “Treatment*Investment amount” regression estimated as an ordinary least squares regression with clustering on the individual as the linear mixed-effect model used in Table S8 did not converge; five observations per participant included in this regression explaining the high n).

Table S18. Non-preregistered robustness tests of Table S9 results adding controls for time of day, year and month of data collection.[#]

	**Dependent variable: Dictator Value**					
**Independent variable**	**Hypothesized sign**	**n**	**Regression coefficient**	**Standard error**	**t/z-value (df)**	**p-value**
Basal testosterone	Negative	979	0.067	0.095	0.707	0.479
Basal testosterone* Basal cortisol	Positive	956	0.118	0.126	0.934 (932)	0.350
Treatment* Basal cortisol	Positive	957	-0.158	0.149	-1.059 (934)	0.290

[#] Ordinary least squares regression for all Dictator Value regressions except the one for "basal testosterone" based on an ordered logistic regression.

Table S19. Non-preregistered robustness tests of Table S10 results adding controls for time of day, year and month of data collection.[#]

	**Dependent variable: Percentage Donated**					
**Independent variable**	**Hypothesized sign**	**n**	**Regression coefficient**	**Standard error**	**t-value (df)**	**p-value**
Treatment	No hypothesis made in the PAP	11892	-1.101	1.507	-0.731 (898)	0.465
Treatment* Price of donating*Trait impulsivity	positive	11856	-0.930	1.405	-0.662 (360)	0.509
Treatment*Price of donating*Treatment belief	Positive	11724	1.465	5.338	0.274 (815)	0.784
	**Dependent variable: Percentage Donated (robustness test)**					
**Independent variable**	**Hypothesized sign**	**n**	**Regression coefficient**	**Standard error**	**t-value (df)**	**p-value**
Treatment	No hypothesis made in the PAP	10680	-0.372	1.588	-0.234 (790)	0.815
Treatment*Price of donating*Trait impulsivity	Positive	10656	-0.709	1.486	-0.477 (321)	0.634
Treatment*Price of donating*Treatment belief	Positive	10548	1.352	5.639	0.240 (736)	0.811

[#] Ordinary least squares regression with clustering of standard errors on the participant for all Percentage Donated regressions (a linear mixed-effect model regression was preregistered for these regressions, but these regressions failed to converge). Every participant is included with 12 observations in the regression explaining the high n.

Table S20. Non-preregistered robustness tests of Table S11 results adding controls for time of day, year and month of data collection.[#]

	**Dependent variable: Willingness to Compete**					
**Independent variable**	**Hypothesized sign**	**n**	**Regression coefficient**	**Standard error**	**t/z-value (df)**	**p-value**
Basal testosterone	Positive	979	0.085	0.107	0.794	0.427
Basal cortisol	Negative	957	0.082	0.077	1.068	0.286
Basal testosterone* Basal cortisol	Negative	956	-0.001	0.030	-0.032 (932)	0.975
Treatment* Basal cortisol	Negative	957	-0.046	0.034	-1.365 (934)	0.173
Treatment* Trait dominance	Positive	990	-0.048	0.030	-1.595 (967)	0.111
	**Dependent variable: Performance Change Score**					
**Independent variable**	**Hypothesized sign**	**n**	**Regression coefficient**	**Standard error**	**t/value (df)**	**p-value**
Basal testosterone	Positive	979	0.100	0.186	0.537 (957)	0.592
Basal cortisol	Negative	957	0.124	0.120	1.033 (935)	0.302
Basal testosterone* Basal cortisol	Negative	956	0.248	0.210	1.183 (932)	0.237
Treatment* Basal cortisol	Negative	957	-0.256	0.229	-1.121	0.263

[#] Ordinary least squares regression for all Performance Change Score regressions. The Willingness to Compete regressions without any interactions are estimated with logistic regression and the regressions with interactions estimated with ordinary least squares.

Table S21. Non-preregistered robustness test of the exploratory analyses in Table S6 that includes "Basal testosterone" as an independent variable. Outliers in "Basal testosterone" (pg/ml >150) excluded.[#]

	Dependent variable: Risk Aversion[&]					
**Independent variable**	**Hypothesized sign**	**n**	**Regression coefficient**	**Standard error**	**t-value (df)**	**p-value**
Basal testosterone	Positive	859	-0.019	0.016	-1.193 (856)	0.233
Basal testosterone* Basal cortisol	Negative	839	0.0056	0.017	0.326 (834)	0.744
	**Dependent variable: Loss Aversion**					
**Independent variable**	**Hypothesized sign**	**n**	**Regression coefficient**	**Standard error**	**t-value (df)**	**p-value**
Basal testosterone	Negative	859	-0.0006	0.07	-0.008 (856)	0.993
Basal testosterone* Basal cortisol	Positive	839	0.037	0.078	0.476 (834)	0.637

[#] Ordinary least squares regression for all Risk Aversion and Loss Aversion regressions.

[&] Note that a higher risk aversion parameter implies less risk aversion and the hypothesized positive sign thus implies that testosterone is hypothesized to decrease risk aversion.

Table S22. Non-preregistered robustness test of the exploratory analyses in Table S7 that includes "Basal testosterone" as an independent variable. Outliers in "Basal testosterone" (pg/ml >150) excluded.[#]

	Dependent variable: Proposer Value					
**Independent variable**	**Hypothesized sign**	**n**	**Regression coefficient**	**Standard error**	**t-value (df)**	**p-value**
Basal testosterone	Positive	897	0.147	0.075	1.955 (894)	0.051
Basal testosterone* Basal cortisol	Negative	876	0.033	0.072	0.463 (871)	0.643
	**Dependent variable: Responder Value**					
**Independent variable**	**Hypothesized sign**	**n**	**Regression coefficient**	**Standard error**	**t/z-value (df)**	**p-value**
Basal testosterone	Positive	897	0.018	0.117	0.154	0.878
Basal testosterone* Basal cortisol	Negative	876	0.0007	0.023	0.031 (871)	0.975

[#] Ordinary least squares regression for all Proposer Value regressions and all Responder Value regressions except the one for "basal testosterone" and Responder Value based on an ordered logistic regression (as that regression does not include any interactions).

Table S23. Non-preregistered robustness test of the exploratory analyses in Table S8 that includes "Basal testosterone" as an independent variable. Outliers in "Basal testosterone" (pg/ml >150) excluded.[#]

	Dependent variable: Investor Value					
**Independent variable**	**Hypothesized sign**	**n**	**Regression coefficient**	**Standard error**	**t-value (df)**	**p-value**
Basal testosterone	Negative	897	-0.220	0.159	-1.382 (894)	0.167
Basal testosterone* Basal cortisol	Positive	876	-0.183	0.157	-1.167 (871)	0.244
	**Dependent variable: Trustee Value**					
**Independent variable**	**Hypothesized sign**	**n**	**Regression coefficient**	**Standard error**	**t-value (df)**	**p-value**
Basal testosterone	Positive	897	-0.01	0.01	-1.026 (894)	0.305
Basal testosterone* Basal cortisol	Negative	876	0.003	0.013	0.238 (871)	0.812

[#] Ordinary least squares regression for all Investor Value regressions and all Trustee Value regressions.

Table S24. Non-preregistered robustness test of the exploratory analyses in Table S9 that includes "Basal testosterone" as an independent variable. Outliers in "Basal testosterone" (pg/ml >150) excluded.[#]

	Dependent variable: Dictator Value					
**Independent variable**	**Hypothesized sign**	**n**	**Regression coefficient**	**Standard error**	**t/z-value (df)**	**p-value**
Basal testosterone	Negative	897	-0.095	0.121	-0.780	0.435
Basal testosterone* Basal cortisol	Positive	876	-0.186	0.149	-1.245 (871)	0.213

[#] Ordinary least squares regression for all Dictator Value regressions except the one for "basal testosterone" based on an ordered logistic regression.

Table S25. Non-preregistered robustness test of the exploratory analyses in Table S11 that includes "Basal testosterone" as an independent variable. Outliers in "Basal testosterone" (pg/ml >150) excluded.#

	Dependent variable: Willingness to Compete					
**Independent variable**	**Hypothesized sign**	**n**	**Regression coefficient**	**Standard error**	**t/z-value (df)**	**p-value**
Basal testosterone	Positive	897	0.125	0.139	0.897	0.370
Basal testosterone* Basal cortisol	Negative	876	0.010	0.039	0.242 (871)	0.809
	**Dependent variable: Performance Change Score**					
**Independent variable**	**Hypothesized sign**	**n**	**Regression coefficient**	**Standard error**	**t-value (df)**	**p-value**
Basal testosterone	Positive	897	0.011	0.224	0.049 (894)	0.961
Basal testosterone* Basal cortisol	Negative	876	0.385	0.264	1.461 (871)	0.144

Ordinary least squares regression for all Performance Change Score regressions. The Willingness to Compete regressions without any interactions are estimated with logistic regression and the regressions with interactions estimated with ordinary least squares.

Table S26. Non-preregistered exploratory analyses of non-linear effects of basal testosterone adding the square of the basal testosterone variable to all preregistered exploratory analyses testing for an association between basal testosterone and the outcome measure.#

Dependent variable	n	Basal T regression coefficient	Standard error	t/z-value (df)	p-value	Square of Basal T regression coefficient	Standard error	t/z-value (df)	p-value
Risk Aversion	935	-0.011	0.012	-0.878 (931)	0.38	-0.002	0.009	-0.236 (931)	0.814
Loss Aversion	935	-0.003	0.052	-0.066 (931)	0.947	0.032	0.03	1.067 (931)	0.286
Proposer Value	979	0.102	0.054	1.895 (975)	0.058	-0.005	0.02	-0.257 (975)	0.797
Responder Valuel	979	-0.134	0.093	-1.444	0.149	0.089	0.047	1.88	0.06
Investor Value	979	0.172	0.141	1.217 (975)	0.224	-0.011	0.086	-0.133 (975)	0.894
Trustee Value	979	-0.007	0.007	-0.952 (975)	0.341	-0.002	0.002	-1.005 (975)	0.315
Dictator Value	979	0.008	0.091	0.092	0.926	-0.016	0.044	-0.368	0.713
Willingness to Compete	979	0.21	0.107	1.953	0.051	-0.026	0.052	-0.495	0.621
Performance Change Score	979	0.054	0.218	0.246 (975)	0.806	0.187	0.159	1.176 (975)	0.24

Ordinary least squares regression for Proposer Value, Investor Value, Trustee Value, Risk Aversion, Loss Aversion, and Performance Change Score; logistic regression for Willingness to Compete; ordered logistic regression for Dictator Value and Responder Value.

Table S27. Non-preregistered exploratory analyses of non-linear effects of basal testosterone adding the square of the basal testosterone variable to all preregistered exploratory analyses testing for an association between basal testosterone and the outcome measure. Outliers in "Basal testosterone" (pg/ml >150) excluded.#

Dependent variable	n	Basal T regression coefficient	Standard error	t/z-value (df)	p-value	Square of Basal T regression coefficient	Standard error	t/z-value(df)	p-value
Risk Aversion	859	-0.012	0.018	-0.642 (855)	0.521	0.025	0.019	1.318 (855)	0.188
Loss Aversion	859	-0.014	0.080	-0.178 (855)	0.859	-0.046	0.089	-0.514 (855)	0.608
Proposer Value	897	0.152	0.087	1.741 (893)	0.082	0.007	0.102	0.064 (893)	0.949
Responder Value	897	-0.003	0.131	-0.02	0.984	-0.024	0.071	-0.346	0.729
Investor Value	897	-0.184	0.182	-1.009 (893)	0.313	0.049	0.148	0.33 (893)	0.742
Trustee Value	897	-0.007	0.011	-0.576 (893)	0.565	0.005	0.006	0.923 (893)	0.356
Dictator Value	897	-0.047	0.135	-0.346	0.730	0.071	0.087	0.814	0.416
Willingness to Compete	897	0.317	0.154	2.056	0.040	0.390	0.190	2.053	0.040
Performance Change Score	897	-0.069	0.238	-0.289 (893)	0.772	-0.106	0.494	-0.214 (893)	0.830

Ordinary least squares regression for Proposer Value, Investor Value, Trustee Value, Risk Aversion, Loss Aversion, and Performance Change Score; logistic regression for Willingness to Compete; ordered logistic regression for Dictator Value and Responder Value.

Table S28. Non-preregistered exploratory analyses testing for an interaction between treatment and basal testosterone in all preregistered exploratory analyses testing for an association between basal testosterone and the outcome measure. All regressions are ordinary least squares regressions.

Dependent variable	n	Treatment* Basal testosterone regression coefficient	Standard error	t-value (df)	p-value
Risk Aversion	935	-0.02	0.025	-0.798 (931)	0.425
Loss Aversion	935	-0.036	0.094	-0.383 (931)	0.702
Proposer Value	979	-0.107	0.111	-0.959 (975)	0.338
Responder Valuel	979	-0.019	0.0346	-0.544 (975)	0.586
Investor Value	979	-0.152	0.247	-0.617 (975)	0.537
Trustee Value	979	-0.013	0.014	-0.938 (975)	0.349
Dictator Value	979	-0.401	0.195	-2.055 (975)	0.401
Willingness to Compete	979	0.029	0.047	0.621 (975)	0.535
Performance Change Score	979	-0.547	0.358	-1.527 (975)	0.127

Table S29. Non-preregistered exploratory analyses testing for an interaction between treatment and basal testosterone in all preregistered exploratory analyses testing for an association between basal testosterone and the outcome measure. All regressions are ordinary least squares regressions. Outliers in "Basal testosterone" (pg/ml >150) excluded.

Dependent variable	n	Treatment* Basal testosterone regression coefficient	Standard error	t-value (df)	p-value
Risk Aversion	859	-0.004	0.032	-0.112 (855)	0.912
Loss Aversion	859	-0.072	0.141	-0.512 (855)	0.608
Proposer Value	897	-0.140	0.151	-0.925 (893)	0.355
Responder Value	897	-0.00005	0.042	-0.001 (893)	0.999
Investor Value	897	0.213	0.333	0.640 (893)	0.522
Trustee Value	897	-0.026	0.020	-1.258 (893)	0.209
Dictator Value	897	-0.394	0.262	-1.504 (893)	0.133
Willingness to Compete	897	-0.023	0.071	-0.321 (893)	0.748
Performance Change Score	897	-0.279	0.476	-0.585 (893)	0.559
